# Forgotten memory storage and retrieval in Drosophila

**DOI:** 10.1038/s41467-023-42753-x

**Published:** 2023-11-07

**Authors:** Chih-Ming Wang, Chun-Yuan Wu, Chen-En Lin, Ming-Chi Hsu, Jing-Chun Lin, Chuan-Chin Huang, Ting-Yu Lien, Hsin-Kai Lin, Ting-Wei Chang, Hsueh-Cheng Chiang

**Affiliations:** 1 Institute of Basic Medical Sciences, College of Medicine, National Cheng Kung University, Tainan, Taiwan, ROC; 2 Brain Research Center, National Tsing Hua University, Hsinchu, Taiwan, ROC; 3 Department of Medicine, National Cheng-Kung University, Tainan, Taiwan, ROC; 4 Department of Pharmacology, College of Medicine, National Cheng-Kung University, Tainan, Taiwan, ROC

**Keywords:** Fear conditioning, Neural circuits

## Abstract

Inaccessibility of stored memory in ensemble cells through the forgetting process causes animals to be unable to respond to natural recalling cues. While accumulating evidence has demonstrated that reactivating memory-stored cells can switch cells from an inaccessible state to an accessible form and lead to recall of previously learned information, the underlying cellular and molecular mechanisms remain elusive. The current study used *Drosophila* as a model to demonstrate that the memory of one-trial aversive olfactory conditioning, although inaccessible within a few hours after learning, is stored in KCαβ and retrievable after mild retraining. One-trial aversive conditioning triggers protein synthesis to form a long-lasting cellular memory trace, approximately 20 days, via creb in KCαβ, and a transient cellular memory trace, approximately one day, via orb in MBON-α3. PPL1-α3 negatively regulates forgotten one-trial conditioning memory retrieval. The current study demonstrated that KCαβ, PPL1-α3, and MBON-α3 collaboratively regulate the formation of forgotten one-cycle aversive conditioning memory formation and retrieval.

## Abstract (web summary)

In fly, one-cycle aversive conditioning-induced choice bias decays in 24 h. Here, authors found the memory of one-cycle aversive conditioning remains in the brain, between KCab and MBON-a3, and is retrievable by a second mild retraining.

## Introduction

Memory that serves to preserve past experiences and affect an animal’s future behavior is the most important cognitive activity; however, memory is not always stable in the brain and constantly fades through a process called forgetting. In contrast to memory formation, which can guide future animal behavior, forgetting weakens stored memory. It offers flexibility for animal behavior to fit the dynamic environment. Emerging evidence has shown that, in addition to memory formation, learning also initiates the forgetting process, which suggests that as early as the beginning of memory formation, memory is bound to decay. However, it remains unclear how the forgetting process interferes with memory formation.

In general, behaviorally, forgetting is considered to be a trained associative response that cannot be induced when an animal encounters recalling cues^1–3^. However, the definition of forgetting has transitioned in the past ten years. Reactivating engram cells in AD mice with APP/PS1 mutation and in protein synthesis inhibition-induced amnesia mice led to the recall of freezing behavior that could not be retrieved by natural recalling cues^4,5^. In addition, 24-hour social memory, which typically cannot be retrieved by recalling cues within 24 h, is also restored after c-fos-labeled neurons in the CA1 region were reactivated^6^. Seminal studies from *Aplysia* show that latent memory can be induced by long-term sensitization stimulation^7–9^. Further study on the transcriptional correlation in *Aplysia* suggests that forgetting could be a retrieval failure^10^. These studies suggest that although no recall behaviors are observed, the memory for conditioned experience has not been erased but is hidden within the system in the form of a silent, inaccessible engram^11,12^, and requires proper stimulation to activate it.

Recent studies on the characterization of silent engram cells suggest that synaptic connectivity is weakened between neurons to cause retrieval failure, and learning-induced physical/chemical changes within cells are partially preserved^4^, although there is also other evidence suggesting that depressed plasticity is not the main contributor to forgetting^13^. Therefore, it remains elusive what cellular activities or signaling pathways triggered by learning are altered during forgetting.

Optogenetics experiments to reactivate learning-activated engram cells have shown recall of the conditioned behaviors^14^, suggesting a population of cells designed to direct animal behavior upon reexperiencing recalling cues. This experimental setting intensely relies on learning-induced IEGs activation, and learning-induced engram cells activation is tracked by IEGs labeling. However, recent studies have proposed the heterogeneity of engram cells, suggesting that the activation of certain IEGs cannot represent all engram cells^15^. A study using fosGFP to label activated cells showed that in the primary sensory cortex, c-fos labeling could not reveal all learning-related neural ensembles^16^ suggesting that not all engram cells display the same characteristics. In addition, the role of inhibitory engram cells in managing memory formation and forgetting has been proposed but has not been experimentally characterized^17^. More studies are needed to reveal the natural properties of different memory cells in managing memory formation.

Experimental psychology has proposed that nonpathological forgetting could be due to interference (active forgetting) and natural decay (passive forgetting)^2,18–20^. Recent studies have found that some active forgetting processes, intrinsic and interference-based forgetting, are regulated by learning-activated dopamine/Rac1 and cdc42 signaling pathways^21–23^. It has been proposed that changing the memory formation signaling pathway to facilitate or delay the forgetting process could manipulate engram cell activity and affect the quality and quantity of stored memory^19^. However, it remains uncertain how the intrinsic and interference-based forgetting processes affect memory trace formation and decay.

Classical olfactory conditioning is a widely used learning paradigm in Drosophila. It has been established that olfactory conditioning training can form short-term memory (STM), middle-term memory (MTM), and long-term memory (LTM). While STM is usually lost within several hours after learning, LTM lasts at least days^24,25^. Traditionally, regular one-cycle olfactory aversive conditioning induces STM and MTM expression, which do not require new protein synthesis, while spaced training (multiple cycles of training with resting intervals) is needed to trigger new protein synthesis to form LTM. Although there are some reports that one cycle of conditioning is able to induce LTM^26–28^, here we focus on a protocol that has been well established to study STM and MTM but no protein synthesis dependent-LTM formation^29^.

Decades of studies have concluded that Kenyon cells (KCs) in the mushroom bodies (MBs) of fruit flies are the primary neurons for learning and memory and can be subdivided into three specific regions, KCγ, KCαβ, and KCα’β’^30^. Accumulated evidence has demonstrated that KCαβ and their downstream output neurons, MBONs, are responsible for LTM retrieval^31–35^, suggesting that LTM is stored within an αβ circuit.

The current study used fruit flies as a model to investigate how “forgotten memory” of one-cycle aversive conditioning is stored in the brain and the cellular mechanism to retrieve it. We found that memory of one-cycle olfactory classical conditioning was retrievable eight days after learning when trained flies experienced a second mild retraining. We confirmed that this mild retraining was not establishing a new memory but recalling previously stored and “forgotten” memory. Importantly, we found that passive forgetting and active forgetting are similar processes, but with different decay rates. Further analysis showed that KCαβ, MBON-α3, and PPL1-α3 behave similarly to engram cells, collaboratively working together to regulate the forgetting process and retrieve forgotten one-cycle olfactory classical conditioning memory. The current study demonstrates a pool of heterogeneous engram-like cells with different activation times and molecular signaling that are activated by learning and require protein synthesis to manage memory storage and retrieval.

## Results

### The memory of one-cycle aversive conditioning is retrievable after eight days

**Fig. 1:**
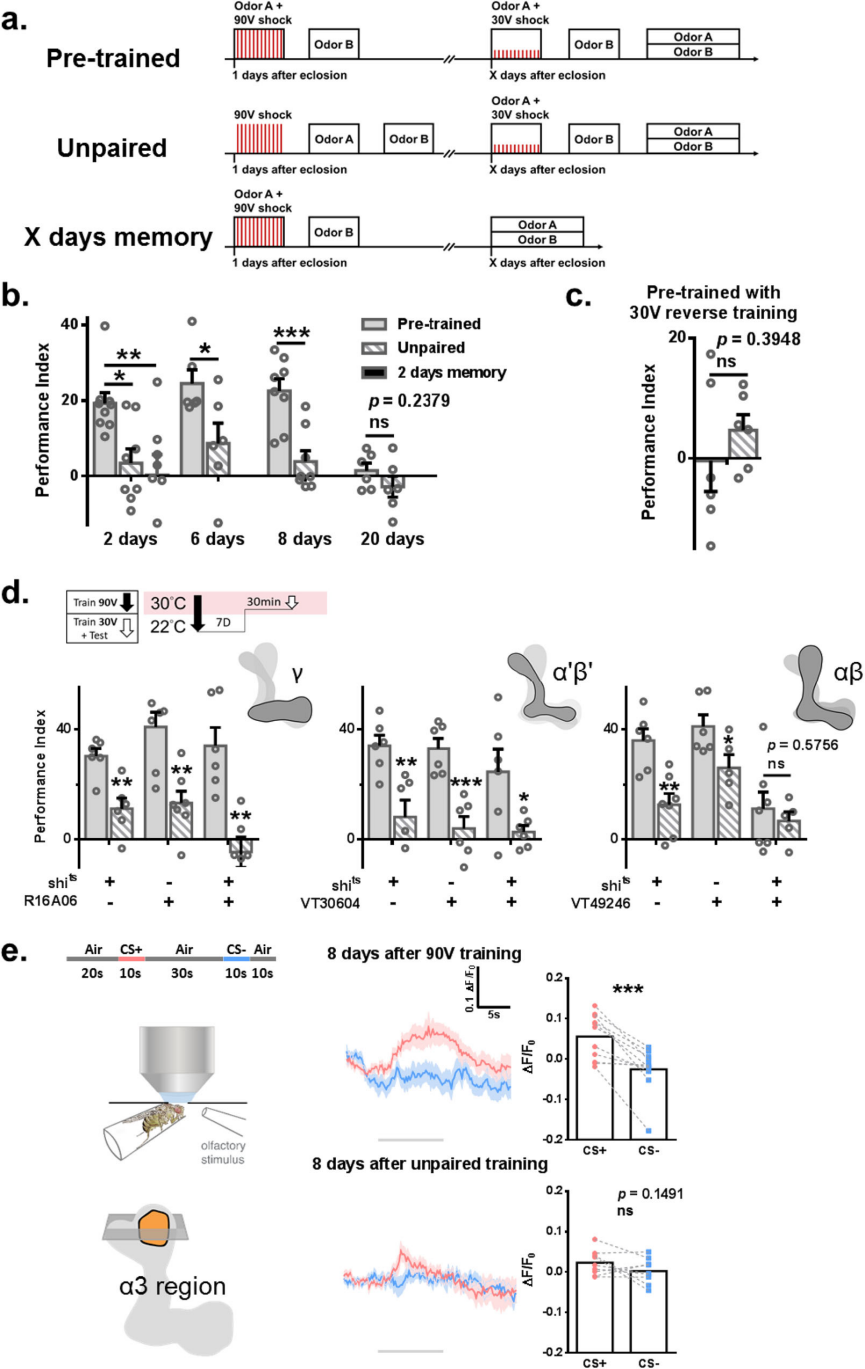
The memory of one-cycle aversive conditioning is retrievable. **a** Experimental protocol. **b** The memory of one-cycle aversive conditioning remained retrievable after mild-stimulation, 30 V training in the pretrained group. There was no significant behavioral performance observed in the unpaired group. There was no retrieved memory found 2 days after 90 V training, 2 days memory. For the 2-day group, statistical comparison was carried out by one-way ANOVA with Dunnett’s post-hoc test. *N* = 9, 8 and 8, *p* = 0.0201, 0.0057. For the other groups, statistical comparison was carried out by two-tailed unpaired *t-*test. *N* = 6 and 6, *p* = 0.0346 for the 6-day group. *N* = 8 and 8, *p* = 0.0006 for the 8-day group. *N* = 6 and 6, *p* = 0.2379 for the 20-day group. **c** No memory performance was found when the CS+ and CS- odors were reversed during mild retraining. *N* = 6 for each group. Statistical comparison was carried out by two-tailed unpaired *t*-test, *p* = 0.3948. **d** Output activity inhibition in KCαβ during the fm-retrieval phase inhibited forgotten memory. *N* = 6, 6, 7, 7, 6, 6; 6, 6, 6, 6, 6, 6; 6, 7, 6, 5, 7, 5. Statistical comparison was carried out by two-tailed unpaired *t*-test, *p* = 0.0022, 0.0017, 0.0012; 0.005, 0.0004, 0.0291; 0.0022, 0.0408, 0.5756 (from left to right). **e** Left: experimental setup. Right: Increased calcium signal from GCaMP7f was found during CS+ exposure in the flies 8 days after 90 V training but not 8 days after unpaired training. The gray bar represents the time of odor delivery and the area for statistical analysis. *N* = 11 and 9. Calcium imaging data (ΔF/F0) were evaluated by two-tailed paired *t*-test, *p* = 0.0005 and 0.1491. ^*^p < 0.05. ^**^p < 0.01. ^***^p < 0.001. In all figures, each value represents the mean ± SEM. Shi^ts^, shibire^temperature-sensitive^. CS +, conditioned stimulus positive. CS-, conditioned stimulus negative.

It has been widely accepted that the memory of regular one-cycle aversive conditioning decays within 24 h; flies do not show any choice bias to avoid the conditioned stimulus (CS+) odor. To understand if such memory remains available and can be retrieved to direct animal behavior, an experimental protocol was set up to address this issue (Fig. 1a). We hypothesized that mild retraining could activate the recalling circuit to retrieve previously formed but forgotten one-cycle olfactory aversive conditioning memory. For simplicity, we called forgotten one-cycle aversive conditioning memory a forgotten memory. Our data showed that mild retraining, 30 V training, retrieved forgotten memory 2, 6, and 8 days but not 20 days after 90 V training (Fig. 1b). Further analysis showed that forgotten memory was very stable between testing days (Supplementary Fig. 1a). Unless mentioned, we used a paradigm with an 8-day interval between the 90 V training and mild retraining. Without mild retraining, forgotten memory could not be retrieved two days after 90 V training (Fig. 1b). CS+ or CS- odor stimuli or electrical shock (E.S.) alone could not retrieve forgotten memory, and pretrained flies showed higher performance than unpaired and naïve flies trained with only 30 V (Supplementary Fig. 1b, c). The forgotten memory could also be established in older flies. We trained older flies, eight days old, and delivered mild retraining five days later (Supplementary Fig. 1d). Under this condition, forgotten memory could still be established with less efficiency. However, the retrieved forgotten memory decayed within 6 h, suggesting that this is short-term memory (Supplementary Fig. 1e). Further studies confirmed that mild retraining retrieves previously stored “forgotten memory” but does not establish a new memory. 1. No or very low behavioral performance was observed with only 30 V training (Supplementary Fig. 1c). 2. No behavioral performance was observed in mild reversal training (Fig. 1c), and the CS+ and CS- odors used in 90 V training were switched in mild retraining. 3. We used VT49246-Gal4 for KCαβ, VT30604-Gal4 for KCα‘β‘, and R16A06-Gal4 for KCγ^36^ to overexpress a temperature-sensitive shibire mutant, shi^ts^, to identify where the forgotten memory was stored. All used transgenic lines were listed in the Table 1. Flies were moved to the restrictive temperature 30 min before mild retraining and testing. For simplicity, we called the first 90 V training as training (90 V-training) and mild retraining plus testing as forgotten memory retrieval (fm-retrieval). Output activity inhibition in KCαβ prohibited forgotten memory retrieval (Fig. 1d). Accumulated evidence has demonstrated that ΚCγ and KCα‘β‘ neurons are responsible for acquisition and consolidation, while KCαβ is responsible for aversive conditioning LTM retrieval^34,37,38^. Therefore, the forgotten memory was likely retrieved from a previously formed memory rather than establishing a new memory. We also confirmed that the electric reactivity of 30 V was similar between pretrained and unpaired flies (Supplementary Fig. 1f). 4. Dopamine signaling is required in KCγ for CS-US association^39^. Consistently, we also found that knocking down Dop1R1, a type 1 dopamine receptor, in neurons decreased behavioral performance (Supplementary Fig. 1g). However, knockdown of Dop1R1 in KCγ four days before the fm-retrieval phase did not block forgotten memory retrieval (Supplementary Fig. 1h), suggesting that new association memory does not contribute to retrieving forgotten memory. 5. To directly show that forgotten memory is stored in αβ neurons, we used GCaMP7f to observe the change in intracellular calcium levels to reflect neural activity. We found that the ratio of the CS+/CS- calcium signal was increased in the α branch of KCαβ in the previously 90 V trained flies, without mild retraining, after eight days (Fig. 1e and Supplementary Fig. 1i). We analyzed the tip of the α region, the α3 compartment, as this region has been used to study the GCaMP response after learning^32,33,40^. There was no difference between the response to CS+ and CS- stimuli in the calyx region in the pretrained flies eight days later, suggesting that forgotten memory is not stored in the calyx region (Supplementary Fig. 1j). This result was repeated when we switched the odor for CS+ and CS- (Supplementary Fig. 1k). The formed calcium trace that responds to the CS+ after learning has also been suggested as the cellular memory trace^32,40^. The long-lasting cellular memory trace after one-cycle aversive conditioning found in the α branch differs from the results of a previous study. Yu et al. showed no calcium signal difference between CS+ and CS- after 24 h^32^. The discrepancy could be due to the sensitivity of GCaMP, different versions of GCaMP, different Gal4 lines used, and different protocols. Altogether, our data showed that one-cycle olfactory aversive conditioning memory has not vanished but is hidden within the neural circuit.

**Table 1:** Materials used in this study

REAGENT or RESOURCE	SOURCE	IDENTIFIER
*Antibodies*		
Mouse monoclonal anti-puromycin 1:200	DSHB	RRID: AB_2619605
Peroxidase AffiniPure Goat Anti-Mouse IgG (H+L) 1:2000	Jackson ImmunoResearch	115-035-003
Rabbit anti-GAPDH 1:50000	GeneTex	GTX100118
*Chemicals, peptides, and recombinant proteins*		
Mineral Oil(heavy)	Sigma-Aldrich	Cat#330760
3-Octanol (99%)	Sigma-Aldrich	Cat#218405
4-Methylcyclohexanol (98%)	Sigma-Aldrich	Cat#153095
Ethyl acetate(99.8%)	Sigma-Aldrich	Cat#270989
Isopentyl acetate(99%)	Alfa Aesar	Cat#B21618
Cycloheximide	Cayman	Cat#14126
Puromycin	Cayman	Cat#13884
Actinomycin D	Cayman	Cat#11421
*Experimental models: Organisms/strains*		
*D. melanogaster: W1118*	From Dr. Zhong Yi	N/A
*D. melanogaster.UAS-Shibire*^*ts*^	From Dr. Zhong Yi	N/A
*D. melanogaster. VT49246-Gal4*	VDRC	VDRC ID_ 205379
*D. melanogaster. VT30604-Gal4*	VDRC	VDRC ID_ 200228
*D. melanogaster. R16A06-Gal4*	BDSC	RRID: BDSC_48709
*D. melanogaster.UAS- Dop1R1 RNAi*	BDSC	RRID: BDSC_62193
*D. melanogaster.UAS- Dop1R2 RNAi*	BDSC	RRID: BDSC_26018
*D. melanogaster.UAS-ricin*^*cs*^	BDSC	RRID: BDSC_38623
*D. melanogaster.c217-GAL4*	BDSC	RRID: BDSC_30827
*D. melanogaster.per-GAL4*	BDSC	RRID: BDSC_7127
*D. melanogaster.cry-GAL4*	BDSC	RRID: BDSC_24514
*D. melanogaster.ser-GAL4*	BDSC	RRID: BDSC_6791
*D. melanogaster.c507-GAL4*	BDSC	RRID: BDSC_30840
*D. melanogaster.c42-GAL4*	BDSC	RRID: BDSC_30835
*D. melanogaster.R11F03-GAL4*	BDSC	RRID: BDSC_48464
*D. melanogaster.GH146-GAL4*	BDSC	RRID: BDSC_91812
*D. melanogaster.repo-GAL4*	BDSC	RRID: BDSC_7415
*D. melanogaster.MB399B-GAL4*	BDSC	RRID: BDSC_68369
*D. melanogaster.MB549C-GAL4*	BDSC	RRID: BDSC_68373
*D. melanogaster.MB542B-GAL4*	BDSC	RRID: BDSC_68372
*D. melanogaster.MB310C-GAL4*	BDSC	RRID: BDSC_68313
*D. melanogaster.MB077B-GAL4*	BDSC	RRID: BDSC_68283
*D. melanogaster.MB011B-GAL4*	BDSC	RRID: BDSC_68294
*D. melanogaster.MB434B-GAL4*	BDSC	RRID: BDSC_68325
*D. melanogaster.MB504B-GAL4*	BDSC	RRID: BDSC_68329
*D. melanogaster.MB080C-GAL4*	BDSC	RRID: BDSC_68285
*D. melanogaster.G0239-GAL4*	BDSC	RRID: BDSC_12639
*D. melanogaster. MB630B-GAL4*	BDSC	RRID: BDSC_68334
*D. melanogaster. MB504B-Gal4*	BDSC	RRID: BDSC_68329
*D. melanogaster. R58E02-Gal4*	BDSC	RRID: BDSC_41347
*D. melanogaster. MB112C-Gal4*	BDSC	RRID: BDSC_68263
*D. melanogaster. MB082C-Gal4*	BDSC	RRID: BDSC_68286
*D. melanogaster. DAL-Gal4*	From Dr. Ann-Shyn Chiang	N/A
*D. melanogaster. Elav-Gal4*	From Dr. Zhong Yi	N/A
*D. melanogaster. UAS-CrebB RNAi*	BDSC	RRID: BDSC_63681
*D. melanogaster. UAS-CrebA RNAi*	BDSC	RRID: BDSC_42562
*D. melanogaster. UAS-orb RNAi*	BDSC	RRID: BDSC_43143
*D. melanogaster. UAS-orb RNAi*	BDSC	RRID: BDSC_64002
*D. melanogaster. Tublin-Gal80TS*	BDSC	RRID: BDSC_7017
*D. melanogaster. CRE-Gal4.AD*	(Yamazaki et al., 2018)	N/A
*D. melanogaster. R44E04-GAL4.DBD*	BDSC	RRID: BDSC_68291
*D. melanogaster. R13F02-Gal4*	BDSC	RRID: BDSC_48571
*D. melanogaster*. UAS-*amn RNAi*	BDSC	RRID: BDSC_25797
*D. melanogaster. UAS-radish RNAi*	BDSC	RRID: BDSC_39920
*D. melanogaster. UAS-octβ2* *R RNAi*	(Wu et al., 2013)	N/A
*D. melanogaster.UAS- Rac1.V12*	BDSC	RRID: BDSC_6291
*D. melanogaster. UAS-cdc42.V12*	BDSC	RRID: BDSC_4854
*D. melanogaster. 20xUAS-jGCaMP7f*	BDSC	RRID: BDSC_80906
*Software and algorithms*		
Fiji	Schindelin et al., 2012	https://imagej.net/software/fiji/
VisiView® 4.4	Visitron Systems GmbH	https://www.visitron.de/index.html

### The neural circuit is for information transmission

**Fig. 2:**
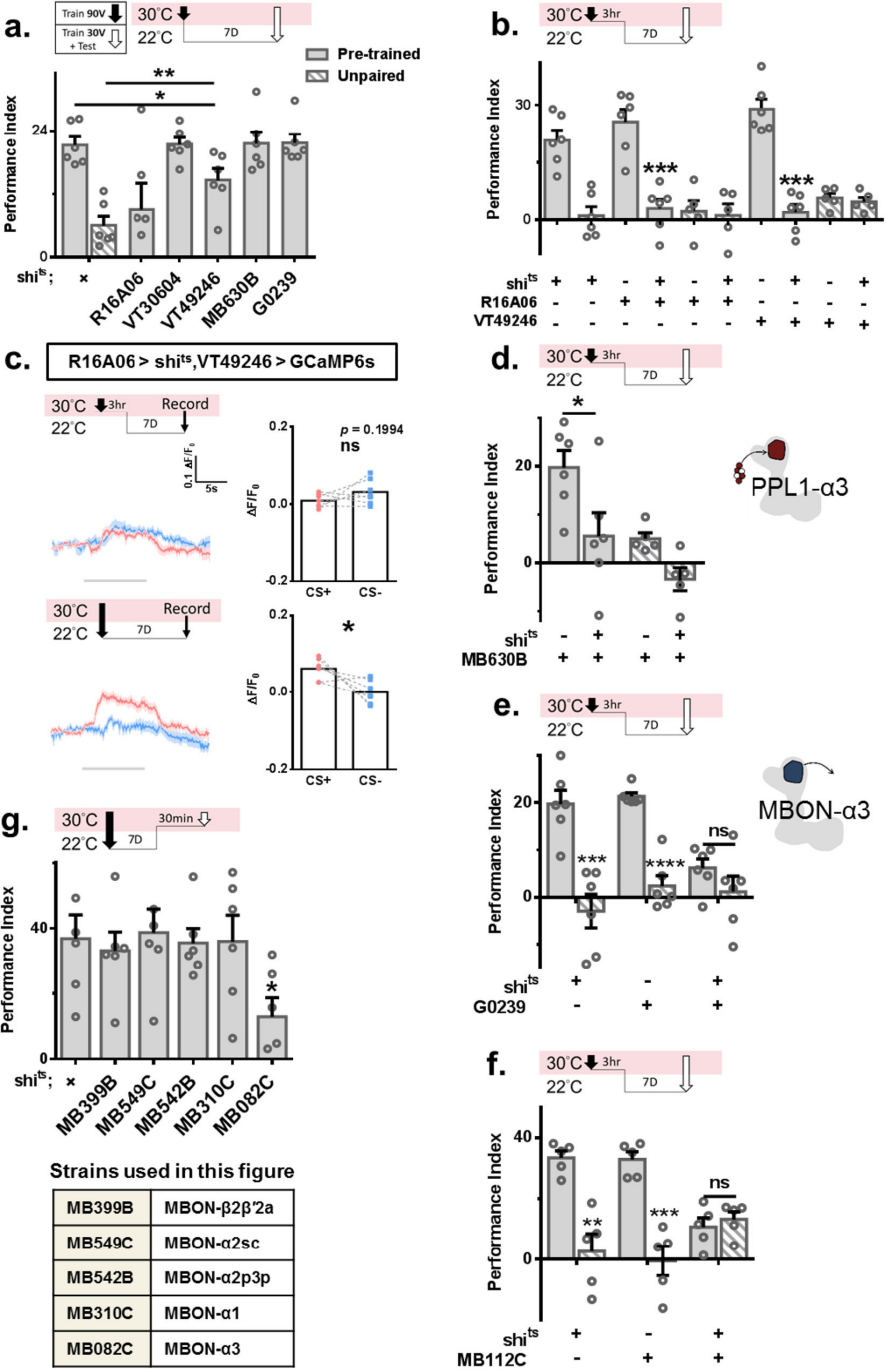
A neural circuit is involved in forming and retrieving forgotten memory. **a** Output activity inhibition in KCγ and KCαβ during 90 V training affected forgotten memory. *N* = 6 for each group. Statistical comparison was carried out by one-way ANOVA with Dunnett’s post-hoc test, *p* = 0.0416, 0.0098. **b,** **d,** **e**, **f** Output activity inhibition in KCγ, KCαβ, PPL1-α3 (MB630B), MBON-γ1pedc > αβ (MB112C) and MBON-α3 (G0239) during 90 V training and 3 h after 90 V training affected forgotten memory. For **b**, *N* = 6, 6, 6, 6, 5, 5, 6, 6, 5, 5, statistical comparison was carried out by one-way ANOVA with Dunnett’s post-hoc test, *p* = 0.0001, 0.0002. For **d**, *N* = 6 for each group, statistical comparison was carried out by two-tailed unpaired *t*-test, *p* = 0.0386. For **e**, *N* = 6 for each group, statistical comparison was carried out by two-tailed unpaired *t*-test, *p* = 0.0006, <0.0001, 0.2125. For **f**, N = 5 for each group, statistical comparison was carried out by two-tailed unpaired *t*-test, *p* = 0.0011, 0.0003, 0.5391. **c** Output activity inhibition of KCγ during the early stage prevented memory trace expression in the α3 region of KCαβ. *N* = 7 for each test group. Calcium imaging data (ΔF/F0) were evaluated by two-tailed paired *t*-test, *p* = 0.1994 and 0.0193. **g** Output activity inhibition in MBON-α3 (MB082C) during mild retraining affected forgotten memory. *N* = 6 for each test group. Statistical comparison was carried out by two-tailed unpaired *t*-test, *p* = 0.0264. ^*^p < 0.05. ^**^p < 0.01. ^***^p < 0.001. ^****^p < 0.0001. In all figures, each value represents the mean ± SEM. Shi^ts^, shibire^temperature-sensitive^. CS +, conditioned stimulus positive. CS-, conditioned stimulus negative.

We were curious how information is processed after one-cycle olfactory aversive conditioning to form forgotten memory. Transgenic flies were moved to restrictive temperatures only during 90 V training. Our data showed damaged forgotten memory in *VT49246-Gal4* > *UAS-Shi*^*ts*^ and *R16A06-Gal4* > *UAS-Shi*^*ts*^ flies (Fig. 2a). These data showed that activation of KCγ and KCαβ is required during acquisition and 90 V-training, to form forgotten memory. The finding of KCαβ involvement is unexpected, as KCαβ has only been suggested to be involved in aversive memory retrieval^34,37,41–43^.

Further studies showed that multiple neurons are involved in the early stage of learning. Transgenic flies were moved to 30 °C during and 3 h after 90 V training. Reduced output activity of KCγ and KCαβ prevented forgotten memory formation (Fig. 2b and Supplementary Fig. 2a). Inhibited KCαβ activity during the early stage produced more damage than in the acquisition stage (Fig. 2a, b). Further analysis showed that early activation of KCγ was crucial for memory trace expression in the α3 region of KCαβ (Fig. 2c). As the activity of KCγ is important for establishing memory, inhibition of KCγ during training jeopardizes learning, and therefore, no “forgotten memory” could be formed. This finding suggests that early training is important to establish an underlying memory circuit to be uncovered later. The activity of PPL1 neurons but not PAM neurons is required during the early stage (Supplementary Fig. 2b). MB504B-Gal4 is for PPL1 neurons, and R58E02-Gal4 is for PAM neurons^44^. PPL1-α3 (MB630B) was required in the consolidation phase but not during acquisition (Figs. 2a, d). Two MBONs are also required during the early stage, MBON-α3 (G0239) and MBON-γ1pedc > αβ (MB112C) (Fig. 2e, f). Further analysis suggested that MBON-α3 is involved in the consolidation phase, as inhibited output activity of MBON-α3 during 90V-training did not affect forgotten memory (Fig. 2a). To search for the downstream circuit of MBNs during the fm-retrieval phase, we looked for the MBONs. Inhibited output activity in *MB082C-Gal4* > *UAS-Shi*^*ts*^ and *G0239-Gal4* > *UAS-Shi*^*ts*^ flies during the fm-retrieval phase blocked forgotten memory retrieval (Fig. 2g and Supplementary Fig. 2c, d). MB082C-Gal4 is for MBON-α3. These results suggested that KCγ and KCαβ are responsible for acquisition and consolidation. As the learned relative odor specificity required to be established by MBON-γ1ped > αβ has been proposed by previous work^42^, we suggest that the activity of MBON-γ1ped > αβ is important to establish odor information for later forgotten memory formation. The forgotten memory is likely hidden in the α3 region of KCαβ and MBON-α3.

**Fig. 3:**
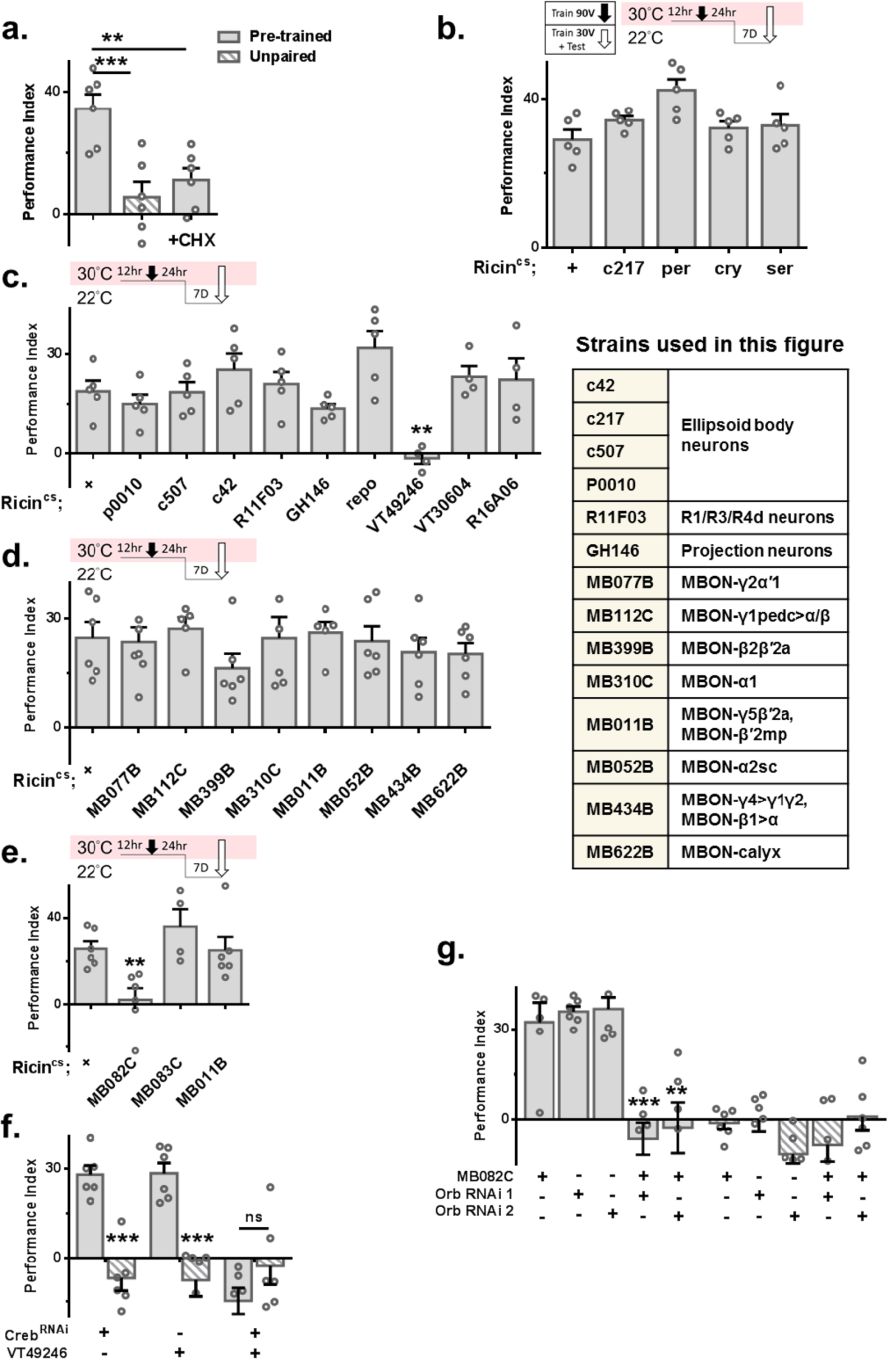
One cycle of aversive conditioning triggers protein synthesis for forgotten memory formation. **a** Flies fed CHX 12 h before and 24 h after 90 V training showed disruption of forgotten memory. *N* = 6 for each test group, statistical comparison was carried out by one-way ANOVA with Dunnett’s post-hoc test, p = 0.0009, 0.0048. **b,** **c,** **d**, and **e**) Transgenic flies were transferred to a 30 °C environment 12 h before and 24 h after 90 V training to abolish protein synthesis. Disruption of forgotten memory was found only in *VT29246-Gal4>Ricin*^*cs*^ and *MB082C-Gal4>Ricin*^*cs*^ flies. For **b**, *N* = 5 for each group, statistical comparison was carried out by two-tailed unpaired *t*-test. For **c**, *N* = 5, 5, 5, 5, 5, 5, 5, 4, 4, 4, statistical comparison was carried out by two-tailed unpaired *t*-test, *p* = 0.0015. For **d**, *N* = 6, 7, 5, 6, 6, 5, 6, 6, 6, statistical comparison was carried out by two-tailed unpaired *t*-test. For **e**, *N* = 6, 6, 4, 6, statistical comparison was carried out by two-tailed unpaired *t*-test, *p* = 0.0042. **f** and **g** Knockdown of CrebB and Orb in adult flies in KCαβ and MBON-α3 abolished forgotten memory, respectively. For **f**, *N* = 6 for each group, statistical comparison was carried out by two-tailed unpaired *t*-test, *p* = 0.0001, 0.0003, 0.1556. For **g**, *N* = 6 for each group, statistical comparison was carried out done by one-way ANOVA with Dunnett’s post-hoc test, *p* = 0.0001, 0.0011. ^*^p < 0.05. ^**^p < 0.01. ^***^p < 0.001. ^****^p < 0.0001. In all figures, each value represents the mean ± SEM. Shi^ts^, shibire^temperature-sensitive^. Orb, oo18 RNA-binding protein. CHX cycloheximide, Per period, Cry cryptochrome, Ser Serrate.

It has been established that protein synthesis is needed for LTM formation but not STM. Although forgotten memory is different from LTM, in terms of retrievability, we hypothesized that to maintain long-lasting forgotten memory, one-cycle olfactory aversive conditioning triggers protein synthesis to support forgotten memory storage. Flies were fed cycloheximide (CHX) 12 h before and 24 h after 90 V training. A subsequent mild retraining and test were delivered three days later. No retrieved forgotten memory was found in treated flies (Fig. 3a). Western blot analysis showed more positive puromycin staining one day after 90 V training (Supplementary Fig. 3a). To reveal where protein synthesis is triggered after one-cycle olfactory aversive conditioning, the temperature-dependent ribosomal toxin RICIN^CS^ was used^45,46^. Transgenic flies were moved to restrictive temperatures 12 h before and 24 h after 90 V training. Forgotten memory was tested eight days later. A failure to retrieve forgotten memory was found only in *MB082C-Gal4* > *UAS-Ricin*^*cs*^ and *VT49246-Gal4* > *UAS-Ricin*^*cs*^ flies (Fig. 3b, c, d, and e). Recent studies have shown that protein synthesis is needed in dorsal–anterior–lateral (DAL) neurons for LTM formation^46^. However, forgotten memory remained retrievable in *DAL-Gal4* > *UAS-Ricin*^*cs*^ flies (Supplementary Fig. 3b). Accumulated studies have shown that cyclic AMP response element-binding protein (CREB)-responsive transcription plays a critical role in LTM formation. In *Drosophila*, the crebA and crebB genes encode CREB family proteins^47–50^. To reveal the role of creb in retrieving forgotten memory, we used RNAi to knock down endogenous crebA and crebB by using Elav-Gal4, a pan-neuronal promoter. Creb was knocked down in adult flies eight days before and four days after 90 V training. The forgotten memory was tested four days after 90 V training. A failure to retrieve forgotten memory was found in *Elav-Gal4* > *UAS-CrebB RNAi* flies under the control of Gal80^ts^ (Supplementary Fig. 3c). However, further analysis showed that knocking down crebB in KCαβ but not in MBON-α3 damaged forgotten memory (Fig. 3f and Supplementary Fig. 3d). Confocal imaging confirmed that after 90 V training, there was more RFP signal in the α3 region (Supplementary Fig. 3e), suggesting that more CREB binds to CRE in KCαβ to express RFP. This experiment used split GAL4, *CRE-p65-AD*, and *R44E04-GAL4 DBD* to drive *UAS-RFP*. Intriguingly, we found that knocking down oo18 RNA-binding protein (orb), a transcription factor that has also been shown to be involved in memory formation^35,51^, in MBON-α3 blocked forgotten memory (Fig. 3g). These data suggested that different proteins are required in KCαβ and MBON-α3 to express/support forgotten memory after one-cycle olfactory aversive conditioning.

### Unconsolidated memory is transformed into forgotten memory

**Fig. 4:**
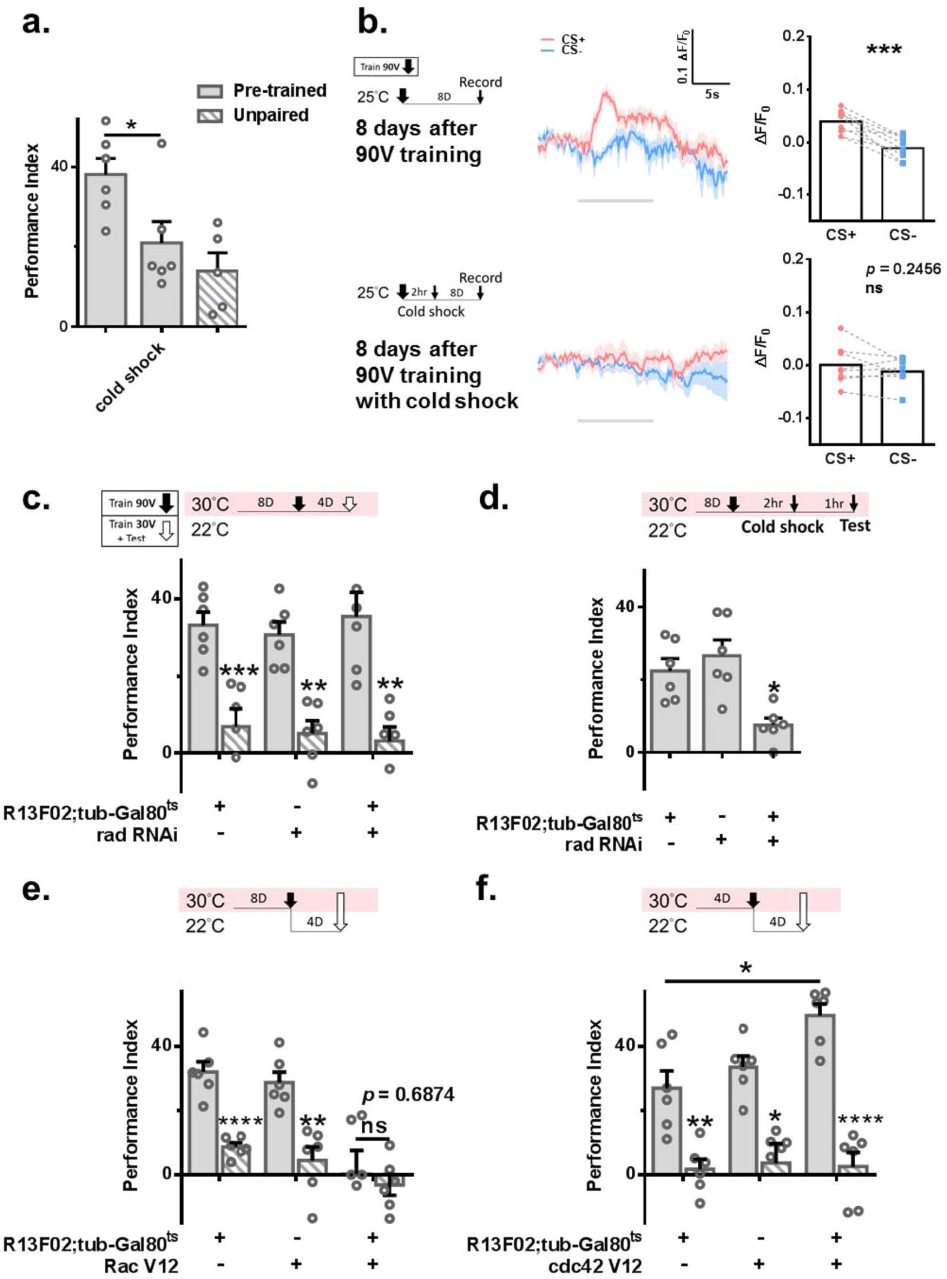
Unconsolidated memory is the main component that forms forgotten memory. **a** 90 V trained flies experienced 2 min of cold-shock 30 min after 90 V training abolished the forgotten memory. *N* = 6, 6, 5. Statistical comparison was carried out by one-way ANOVA with Dunnett’s post-hoc test, *p* = 0.0376. **b** 90 V trained flies that experienced 2 min of cold shock 2 h after 90 V training prevented the formation of a cellular memory trace. *N* = 9 and 8. Calcium imaging data (ΔF/F0) were evaluated by two-tailed paired *t*-test, *p* = 0.0002 and 0.2456. **c** Knocking down radish in adult MBNs for 8 days before and 4 days after 90 V training did not affect forgotten memory. Mild retraining was performed 4 days after 90 V training. *N* = 6 for each group. Statistical comparison was carried out by two-tailed unpaired *t*-test, *p* = 0.0001, 0.0011, 0.0002, 0.0012. **d** Knockdown of radish in adult MBNs for 8 days reduced ARM expression. *N* = 6 for each group. Statistical comparison was carried out by one-way ANOVA with Dunnett’s post-hoc test, *p* = 0.0127. **e**, **f** Overexpressed Rac1 V12 and cdc42 V12 in MBNs disruption and enhanced forgotten memory, respectively. Rac1 V12 was expressed for 8 days before 90 V training. cdc42 V12 was expressed for 4 days before 90 V training. *N* = 6 for each group. For **e**, statistical comparison was carried out by two-tailed unpaired *t*-test, *p* < 0.0001, *p* = 0.001, 0.6874. For **f**, statistical comparison was carried out by one-way ANOVA with Dunnett’s post-hoc test, *p* = 0.002, 0.0301, 0.0387, *p* < 0.0001. ^*^p < 0.05. ^**^p < 0.01. ^***^p < 0.001. ^****^p < 0.0001. In all figures, each value represents the mean ± SEM. Rad radish, Tub tubulin.

In Drosophila, unconsolidated memory (anesthesia-sensitive memory) decays within 6–8 h, while consolidated memory (anesthesia-resistant memory) is formed gradually after learning and can last 24 h^29,52,53^. We examined the role of amnesiac (amn) in forgotten memory. A previous study showed that amn is important for 3 h memory formation^54^. Our results showed that knocking down amn in adult KCαβ did not affect forgotten memory retrieval (Supplementary Fig. 4a). We also confirmed memory damage in *R13F02-Gal4>amn RNAi* flies under the control of Gal80^ts^ (Supplementary Fig. 4b). R13F02-Gal4 is for whole mushroom body neurons. To understand which memory is for forming forgotten memory and is retrieved under our protocol, we cold-shocked the flies 30–40 min after 90 V training for 2 min to disrupt any unconsolidated memory. Under this condition, no forgotten memory was observed retrieved (Fig. 4a). Consistently, no memory trace was found in the α3 region of KCαβ after cold-shock treatment eight days after 90 V training; we applied cold shock 2 h after 90 V training for 2 min (Fig. 4b). Radish is important for ARM formation in fruit flies^52^. Knocking down radish in MBNs, *R13F02-Gal4* > *UAS-radish RNAi*, for eight days before 90V-training did not damage forgotten memory (Fig. 4c). We also confirmed that flies with radish RNAi expression showed ARM deficits (Fig. 4d). A recent study has demonstrated that octopamine signaling also regulates ARM formation^55^. Knockdown of the octopamine receptor OCTβ2 R in MBNs, *R13F02-Gal4* > *UAS-octβ2* *R RNAi*, for eight days before 90V-training did not damage forgotten memory (Supplementary Fig. 4c). To further strengthen our finding that the retrieved forgotten memory in our protocol is from ASM, we examined the role of cdc42 and Rac1. Recent studies have shown that overexpression of Rac1 V12, a constitutive form of Rac1, and cdc42 V12, a constitutive form of cdc42, damages ASM and ARM, respectively^21,23^. Overexpression of Rac1 V12 was observed during 90V-training but not after inhibition of forgotten memory (Fig. 4e and Supplementary Fig. 4d). In contrast, overexpression of cdc42 V12 during 90V-training promoted forgotten memory retrieval (Fig. 4f). As overexpression of cdc42 V12 has been shown to damage ARM maintenance, this study further suggests that disruption of ARM benefits forgotten memory formation. Altogether, we concluded that the forgotten memory we retrieved is mainly from previous ASM decay.

### Formation of the cellular memory trace in KCαβ and MBON-α3

**Fig. 5:**
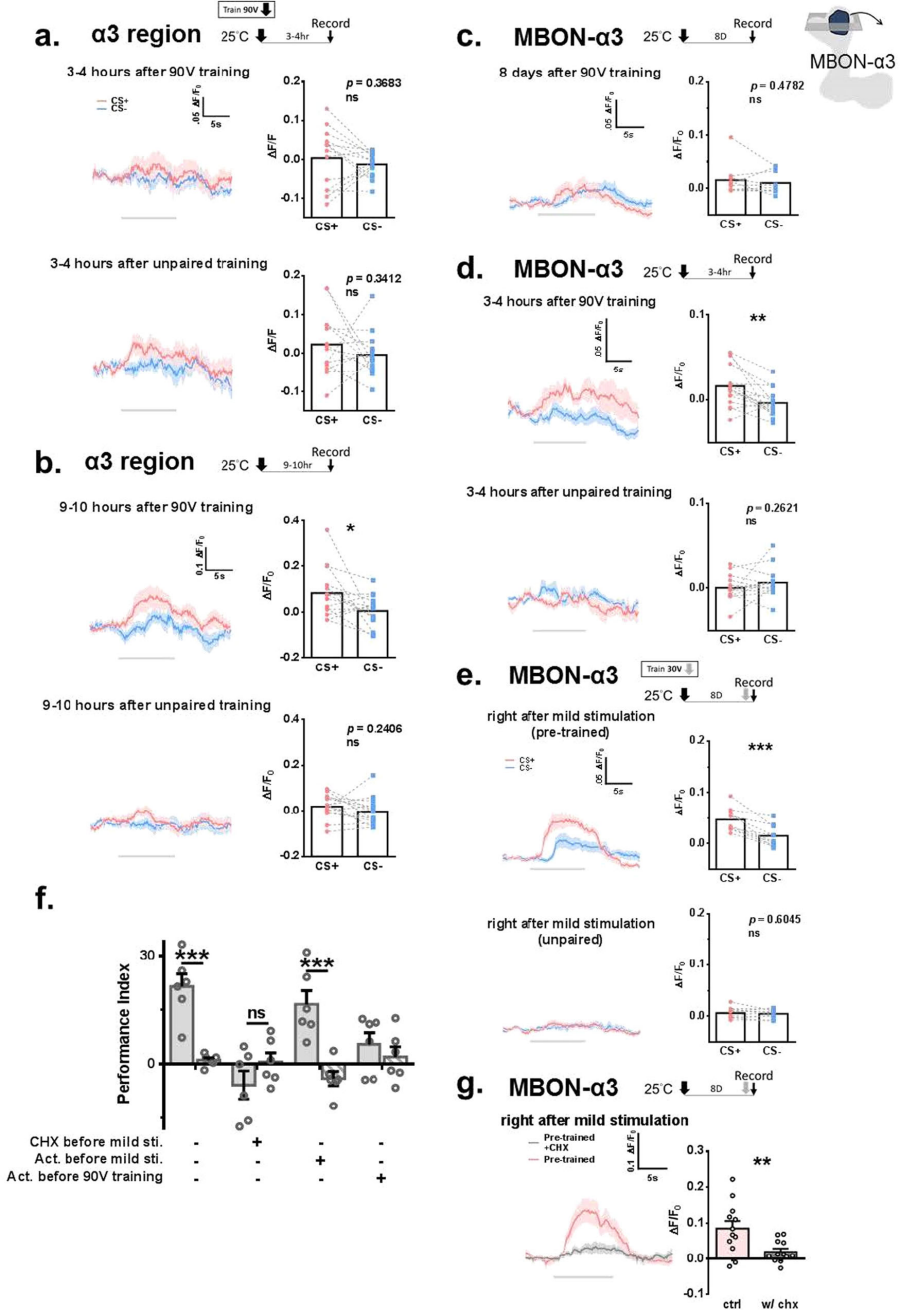
Cellular memory trace formed in the α3 region of KCαβ and MBON-α3 after training. Increased calcium signal during CS+ odor exposure in the α3 region of KCαβ 9–10 h (**b**) but not 3–4 h (**a**) after 90 V training. For both **a** and **b**, *N* = 14 in each group, and the statistical comparisons were carried out by two-tailed paired *t*-test, *p* = 0.3683, 0.3412, 0.0424, 0.2406. (from top to bottom) **c** There was no increased calcium signal during CS+ odor exposure in MBON-α3 8 days after training. *N* = 10. Statistical comparison was carried out by two-tailed paired *t*-test, *p* = 0.4782. **d** Increased calcium signal during CS+ odor exposure in MBON-α3 3–4 h after 90 V-training. *N* = 16 for the paired group, and *N* = 15 for the unpaired group. Statistical comparisons were carried out by two-tailed paired *t*-test, *p* = 0.0036 and 0.2621. **e** Increased calcium signal during CS+ odor exposure in MBON-α3 after mild retraining in the pretrained group. *N* = 10 for each group. Statistical comparisons were carried out by two-tailed paired *t*-test, *p* = 0.0005 and 0.6045. **f** Flies fed CHX but actinomycin D (act.) (5 µg/ml) 24 h before mild retraining abolished forgotten memory. For the before 90 V training group, act. was applied 12 h before and 24 h after 90 V training. *N* = 6 for each group. Statistical comparisons were carried out by two-tailed paired *t*-test, *p* = 0.0002, 0.1999, 0.0007, 0.4316. **g** CHX treatment 24 h before mild retraining decreases the calcium response during CS+ exposure. The data were reanalyzed from Figure S5f, in order to emphasize the difference between groups; experiments were performed at the same time. *N* = 12 in each group. Statistical comparison was carried out by two-tailed unpaired *t*-test, *p* = 0.0085. ^*^p < 0.05. ^**^p < 0.01. ^***^p < 0.001. In all figures, each value represents the mean ± SEM. The *N* values in **a,** **b,** **c,** **d,** **e**, and **g**) represent the number of flies recorded in each experiment, whereas the *N* value in **f**) represents the batches of flies trained and tested in the behavioral assay in each group. CHX, cycloheximide. Act, actin. CS + conditioned stimulus positive, CS- conditioned stimulus negative.

To date, we have shown that KCαβ and MBON-α3 are involved in forgotten memory formation. We decided to further characterize the cellular memory trace formation in KCαβ, and MBON-α3. 90 V-training formed a cellular memory trace in the α3 region of KCαβ 9–10 h but not 3–4 h later (Fig. 5a, b). This formation time is similar to that in a previous study^32^. The cellular memory trace could also be found one day after 90 V-training (Supplementary Fig. 5a). Although no cellular memory trace was found in MBON-α3 8 days after 90 V-training (Fig. 5c), the cellular memory trace was observed 3–4 h after 90 V training (Fig. 5d). MBON-α3 in naïve flies responded to OCT and MCH (Supplementary Fig. 5b). Further analysis showed that the cellular memory trace in MBON-α3 decays within 24 h after 90 V training (Supplementary Fig. 5c). The time course to form the cellular memory trace in MBONs in our study is similar to the previous study in MB-V2^56^. However, we found that the cellular memory trace was quickly formed in MBON-α3 after mild retraining in the pretrained group (Fig. 5e). These data explain why retrieved forgotten memory could instantly affect an animal’s behavior. A quickly formed cellular memory trace was not observed after 90 V training (Supplementary Fig. 5d). The CS + /CS- ratio in MBON-α3 was higher after mild retraining in the pretrained flies, suggesting that pretraining facilitates MBON-α3 to differentiate between CS+ and CS- stimuli (Supplementary Fig. 5e). Drosophila orb is a member of CPEB1 of the CPEB protein^57^. CPEB has been shown to bind mRNA and regulate mRNA translation^58^. Our previous finding that orb is needed in MBON-α3 to regulate forgotten memory led us to hypothesize that, in addition to protein synthesis, 90 V training induced mRNA production and storage in MBON-α3. Untranslated mRNA in MBON-α3 is quickly translated via orb regulation to produce the necessary proteins to form memory traces after mild retraining. Actinomycin, a transcriptional inhibitor, and CHX, a translational inhibitor, were used to confirm our hypothesis. Both drugs were used 24 h before the fm-retrieval phase. CHX but not actinomycin application during the fm-retrieval phase blocked forgotten memory expression (Fig. 5f). Consistently, CHX treatment 24 h before mild retraining decreased the response to CS+ stimulation in MBON-α3 (Fig. 5g and Supplementary Fig. 5f). We also confirmed that actinomycin treatment 12 h before training blocked forgotten memory expression^59–62^ (Fig. 5f).

### Simultaneous PPL1-α3 inhibition and KCαβ activation lead to recall of forgotten memory

**Fig. 6:**
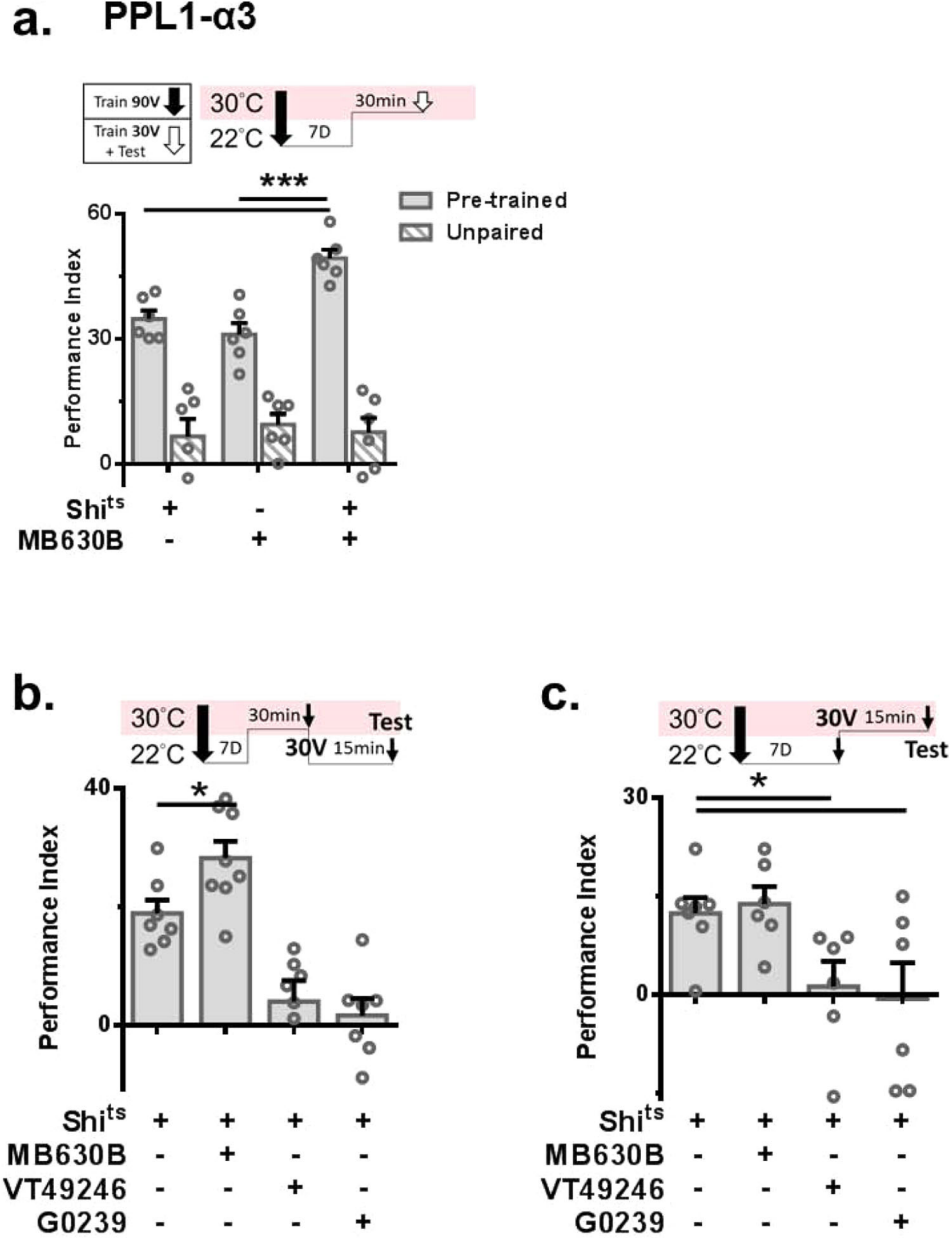
PPL1-α3 negatively regulates forgotten memory retrieval. **a** Output activity inhibition of PPL1-α3 (MB630B) during mild retraining promoted forgotten memory retrieval. *N* = 6 for each group. Statistical comparisons were carried out by one-way ANOVA with Dunnett’s post-hoc test, *p* = 0.001, 0.0001. **b**, **c** Output activity inhibition of KCαβ and MBON-α3 during the retraining and retrieval phases disrupted forgotten memory retrieval, while inhibition of PPL1-α3 only during the retraining phase promoted forgotten memory performance. For **b** *N* = 7, 8, 7, 7 (from left to right). Statistical comparisons were carried out by two-tailed unpaired *t*-test, *p* = 0.0269. For **c** *N* = 7, 6, 6, 6(from left to right). Statistical comparisons were carried out by two-tailed unpaired *t*-test, *p* = 0.0284, 0.0423. ^*^p < 0.05. ^**^p < 0.01. ^***^p < 0.001. In all figures, each value represents the mean ± SEM. The N values in this figure represent the batches of flies trained and tested in the behavioral assay in each group. Shi^ts^, shibire^temperature-sensitive^.

Different specific Gal4 lines were used to examine the role of PPL1 neurons in forgotten memory formation. Our results showed that instead of reducing forgotten memory retrieval, output activity inhibition of PPL1-α3 during the fm-retrieval phase promotes forgotten memory retrieval. This result was confirmed using two PPL1-α3 Gal4 lines, MB060B and MB630B (Fig. 6a and Supplementary Fig. 6). To further dissect the role of the identified memory regulatory neurons, we divided mild retraining into the “retraining phase”, 30 V-paired training, and “retrieval phase” testing. Inhibition of KCαβ and MBON-α3 during the retraining and retrieval phases disrupted forgotten memory retrieval, while inhibition of PPL1-α3 during only the retraining phase promoted forgotten memory expression (Fig. 6b, c). These findings further revealed two different roles of PPL1-α3 in regulating forgotten memory formation and retrieval. While PPL1-α3 activity is required at an early stage to form forgotten memory, the activity of PPL1-α3 during mild retraining negatively regulates forgotten memory retrieval. We also confirmed that there was no effect on forgotten memory formation in permissive temperature conditions in *MB630B-Gal4* > *UAS-shi*^*ts*^*, G0239-Gal4* > *UAS-shi*^*ts*^, and *VT49246-Gal4* > *UAS-shi*^*ts*^ flies (Supplementary Fig. 7).

### The cellular memory trace decays quickly in active forgetting

**Fig. 7:**
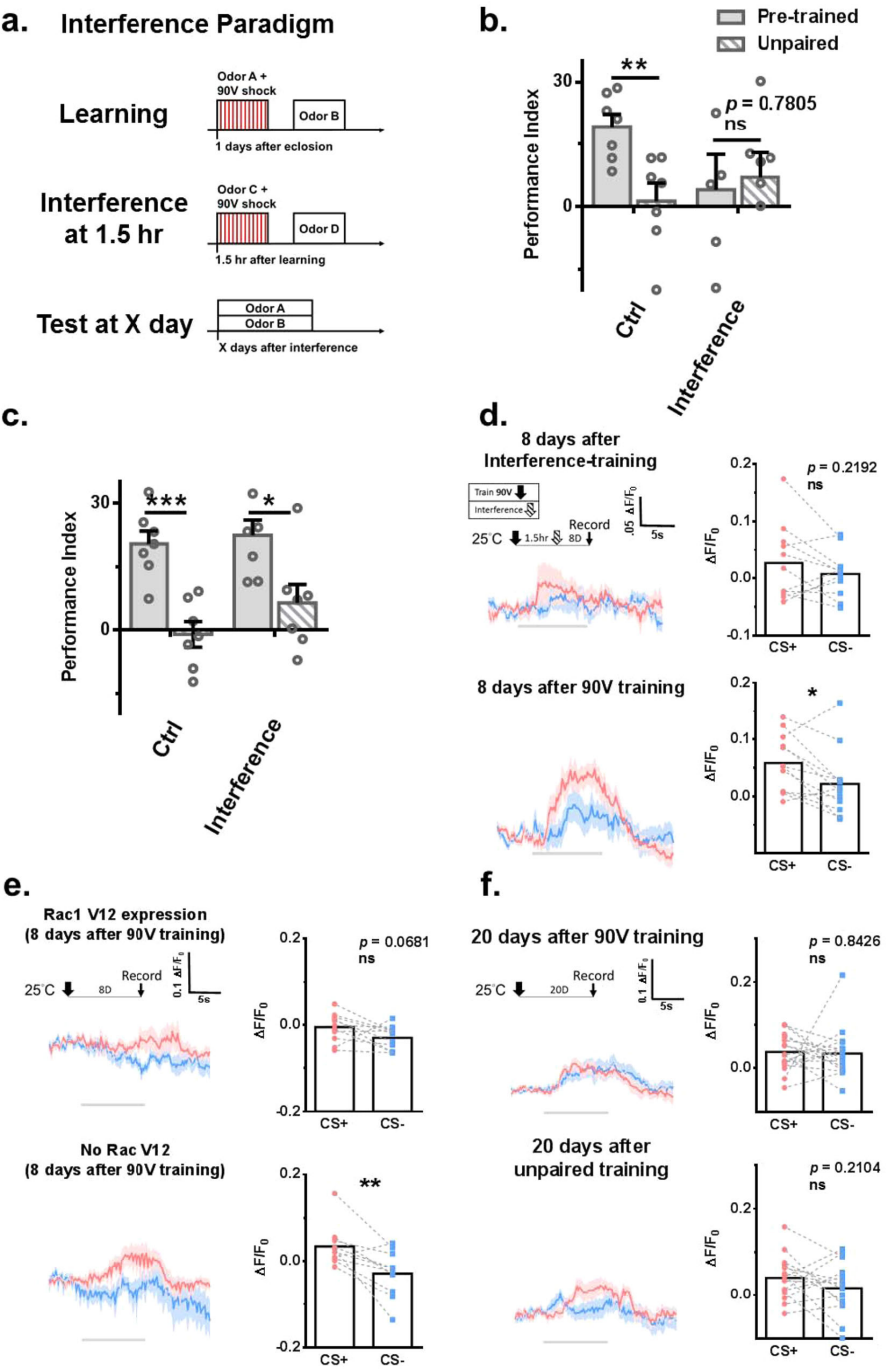
Interference training promotes the decay of cellular memory traces. **a** Experimental protocol. The forgotten memory could be retrieved with mild retraining 3 days (**c**) but not 8 days (**b**) after interference training. For **b**, *N* = 7 in each group. Statistical comparisons were carried out by two-tailed unpaired *t*-test, *p* = 0.0047 and 0.7805. For **c**, *N* = 7 in each group. Statistical comparisons were carried out by two-tailed unpaired *t*-test, *p* = 0.0003 and 0.0162. **d** There was no observed cellular memory trace 8 days after interference training. *N* = 11 and *N* = 12(top to bottom). Statistical comparisons were carried out by two-tailed paired *t*-test, *p* = 0.2192 and 0.0190. **e** There was no observed cellular memory trace in Rac1 V12 overexpression flies 8 days after 90 V training. Rac1 V12 was expressed 8 days before 90 V training. *N* = 11 and *N* = 10(top to bottom). Statistical comparisons were carried out by two-tailed paired *t*-test, *p* = 0.0681 and 0.0054. **f** There was no observed cellular memory trace 20 days after 90 V training in the pretrained group. *N* = 18 and *N* = 15(top to bottom). Statistical comparisons were carried out by two-tailed paired *t*-test, *p* = 0.8426 and 0.2104. ^*^p < 0.05. ^**^p < 0.01. ^***^p < 0.001. In all figures, each value represents the mean ± SEM. The *N* values in **b** and **c** represent the batches of flies trained and tested in the behavioral assay in each group, whereas the *N* values in **d,** **e**, and **f**) represent the number of flies recorded in each experiment. CS +, conditioned stimulus positive. CS- conditioned stimulus negative, Ctrl control.

Interference-based forgetting has been demonstrated as an active forgetting process regulated by Rac1 signaling^21^. We set up a protocol to study the effect of interference (Fig. 7a). Although our data showed that no forgotten memory was retrieved eight days after interference training (Fig. 7b), forgotten memory was retrieved three days later (Fig. 7c). Consistently, no cellular memory trace was observed in the α3 region of KCαβ 8 days after interference-based forgetting (Fig. 7d). These data suggest that forgotten memory quickly decays after interference. Consistently, we found no formed cellular memory trace in Rac1 V12 flies eight days after 90 V training (Fig. 7e). These results led us to re-examine the existence of a cellular memory trace and found no cellular memory trace 20 days after 90 V training (Fig. 7f). The disappearance of the cellular memory trace 20 days after learning suggests that the memory trace could decay passively over time. These studies further imply that forgotten memory in active forgetting is retrievable similarly to passive forgetting, but cellular memory trace decay is much faster in active forgetting.

## Discussion

**Fig. 8:**
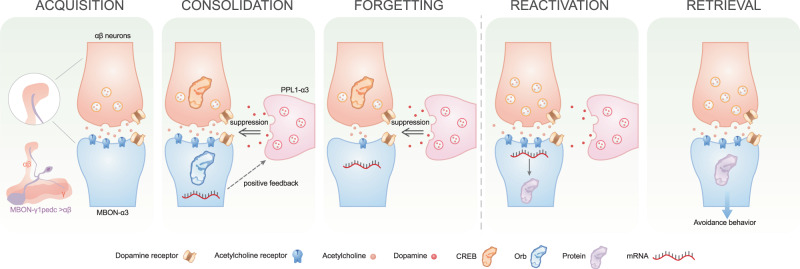
Proposed forgotten memory formation and retrieval. During acquisition and consolidation, 90 V training activates the neural circuit, including KCαβ, MBON-α3, and PPL1-α3. The cellular pathways of CREB and orb are activated in KCαβ and MBON-α3, respectively. The corresponding cellular memory trace is formed in KCαβ and MBON-α3 with different time courses and decay rates. Therefore, the memory of one-cycle aversive conditioning is stored between these two neurons in the early stage. We speculate, dot line, that activated MBON-α3 activates PPL1-α3 to release dopamine to negatively regulate KCαβ and MBON-α3 activity. Forgetting process: released dopamine from PPL1-α3 via dop1R2 to affect the synaptic connection between KCαβ and MBON-α3. Since the cellular memory trace has vanished in MBON-α3 within 24 h, the memory of one-cycle aversive conditioning is only stored in KCαβ. However, the mark of one-cycle conditioning was left in MBON-α3 as mRNA accumulation. During retraining, mild retraining 1) restores and enhances the synaptic connectivity between KCαβ and MBON-α3 and 2) reduces the activity of PPL1-α3. Activated MBON-α3 translates accumulated mRNA to form stable cellular memory. During retrieval, since the positive force, the synaptic connectivity between KCαβ and MBON-α3 is stronger than the negative force, inhibition from PPL1-α3, the memory is retrieved. Passive decay: if there is no similar stimulation in the future to strengthen the activity of KCαβ, the memory would eventually decay, and the memory disappears. Active decay: if there is interference activity from the environment to interfere with memory formation and storage, memory decay will be facilitated. The experience of interference would not affect the formation of memory traces but facilitate memory trace decay. Part of images were Adapted from “Synaptic Cleft with Signaling Molecules (Layout, Horizontal)”, by BioRender.com (2022). Retrieved from https://app.biorender.com/biorender-templates.

The current study reveals how PPL1-α3, KCαβ, and MBON-α3 collaborate to regulate and store “forgotten memory” (Fig. 8). One-cycle aversive conditioning memory is “hidden” in KCαβ and is retrievable with proper stimulation, even after the trained animal is not responding to the recalling cue. Based on our behavioral study, we speculate that PPL1-α3 could negatively regulate forgotten memory retrieval, while KCαβ positively regulates MBON-α3 activity. Mild retraining is a reminder to strengthen the connection between KCαβ and MBON-α3 and activates local translation in MBON-α3 to recover the memory trace. This simple regulatory mechanism ensures that the previously acquired memory is activated only when an animal encounters a similar stimulation.

We hypothesize that at least two different regulatory mechanisms simultaneously conduct a one-cycle aversive conditioning forgetting process. 1) PPL1-α3 releases dopamine to activate dop1R2 in KCαβ and MBON-α3 to weaken synapse connectivity. 2) There is a quick decay of the memory trace in MBON-α3. Training-induced memory trace formation in MBON-α3 decays within 24 h. The memory trace decay in MBON-α3 is regulated passively (time-regulated) and actively (dopamine-regulated). Some recent studies and the current study both showed increased calcium activity during CS+ exposure in MBON-α3^35,63^. Other work showed a decreased calcium response^64^. This discrepancy could be due to the experimental setup, training protocol, status of flies, and different reporter lines used, as discussed in other work^64^.

Accumulated evidence has established that learning-induced engram cell activation is important for memory formation^65,66^. It has been demonstrated that some KCαβ neurons are LTM engram cells^67^. There are three criteria for engram cells: 1) learning-induced activation, 2) learning induces a long-lasting chemical/physical change, and 3) activation is required during recalling^11^. The current studies suggested that the function of KCαβ, PPL1-α3, and MBON-α3 in conducting memory formation and forgetting is similar to the function of engram cells. 1, Early activation of KCαβ, PPL1-α3, and MBON-α3 at different time points during and 3 h after 90 V training suggests that cell-cell communication for memory establishment and storage is activated at a very early stage. Although KCαβ is needed during acquisition and afterward, more sever deficit was found after acquisition, we consider KCαβ is not for learning but mainly in storage. Cervantes-Sandoval et al., proposed a system-like consolidation in fruit fly^34^. In their mode, memory traces are shifted from other MBNs to KCαβ and LTM is only in KCαβ^34^. Our functional cellular imaging is supporting this conclusion and consistent with previously work^32^. We speculated the activation of KCαβ is one of the reasons to promote protein synthesis for later memory storage. 2, Training induces new protein synthesis. The role of protein synthesis induced by one-cycle aversive conditioning has yet to be studied for memory formation. Our data showed that protein synthesis inhibition in KCαβ and MBON-α3 suppressed forgotten memory and diminished the memory trace, suggesting that one-cycle training-induced protein synthesis is important for memory retrieval and the degree of memory trace formation. This conclusion is also consistent with findings in mice^4^. We hypothesize that stronger stimulation causes more protein synthesis, higher quality and quantity and easier memory retrieval. 3, Training induces long-lasting changes. A stable memory trace was found in KCαβ. Although the memory trace of MBON-α3 quickly decayed within 24 h, the existence of learning-induced mRNA accumulation suggests a long-term effect of one-cycle aversive conditioning in MBON-α3. mRNA accumulation in the synapses after stimulation for later rapid local translation to support synaptic plasticity has been proposed as cellular tagging^68,69^. Although the current study did not identify what changes in PPL1-α3 after one-cycle training, the possibility of long-lasting change could not be excluded, for example, strengthening the connection with KCαβ and MBON-α3. *4*, Inhibited output activity of KCαβ and MBON-α3 blocks forgotten memory recall. Decreased PPL1-α3 facilitated forgotten memory retrieval, which also suggests that PPL1-α3 plays an essential role in regulating forgotten memory. Disinhibition to facilitate memory retrieval has been demonstrated in other animal models^17,70^. Currently, we do not know how many neurons activated during acquisition are also activated during retrieval, especially for KCαβ. The results of our study partially support the idea that the heterogeneity of engram cells activated during learning works together to regulate memory expression and forgetting. We did not find a role for KCα’β’ in our study. It could be that either the formed memory in KCα’β‘ vanished during forgetting, or our behavioral protocol could not retrieve the forgotten memory from KCα’β’.

It has been proposed that inhibitory plasticity, mainly from GABAergic input, on engram cells balances the excitatory plasticity and keeps the engram cells in a quiescent state, resulting in behavioral habituation^17,71^. Similar to GABAergic neurons, PPL1-α3 release dopamine to decrease the sensitivity of KCαβ to respond to CS+ vs. CS- stimuli to reduce forgotten memory retrieval. This finding seems to contradict the finding that decreased PPL1-α3 activity during the early stage blocked forgotten memory. There are at least two scenarios to reconcile these findings. 1) The memory of one-cycle aversive learning is gradually “hidden” and unretrievable. Dopamine released by PPL1-α3 promotes the forgetting process. Reduced PPL1-α3 activity at the early stage inhibits the forgetting process. Thus, no “forgetting memory” could be recalled after eight days. This hypothesis is supported by the recent finding that posttraining activity of dopaminergic neurons releases dopamine to activate dop1R2 to conduct the forgetting process^72^. At the fm-retrieval phase, inhibited PPL1-α3 activity reduces forgetting and promotes memory retrieval. 2) The activity of PPL1-α3 is required for memory storage. Although we could not exclude this possibility, this explanation is unlikely, as it has been shown that the activation of PPL1-α3 disrupts memory consolidation^73^.

Our data are consistent with the previous finding that learning induces memory formation and forgetting processes^21,72^. The one-cycle aversive conditioning activates KCαβ and PPL1-α3 suggesting that both excitatory (KCαβ-MBON-α3) and inhibitory (PPL1-α3-KCαβ) signaling are activated by learning. This finding implies that the weight of the synaptic strength between excitatory circuits and the input of inhibitory signaling determines memory decay. We hypothesized that at least two inhibitory signaling pathways affect the forgetting process: weakening the memory trace in KCαβ and the synaptic connectivity between KCαβ and MBON-α3.

The current study further advances our understanding of the different characteristics of KCαβ and MBON-α3 with different molecular pathways, creb v.s. orb, involvement. Although we do not know what causes these signaling differences, we speculate that they may reflect the stability of memory traces. The role of orb in MBON-α3 to support memory expression is consistent with other work^31^. Importantly, a similar function of orb in regulating mRNA translation has been reported in different works^57,74,75^. It would be of great interest to further characterize the detailed molecular mechanism in regulating the formation of different memory traces in different cells in different experimental settings.

The current study reveals several “memory proteins” in forgotten memory formation and retrieval. 1, It has been established that amnesiac is important for MTM. *Amn* mutation and knockdown damages MTM^24,54,76,77^. Our findings suggest that amn is mainly for 3 h memory retrieval rather than memory storage and formation. Although we could not exclude the possibility that STM and MTM are two independent memories and our protocol could not reveal MTM forgetting, given the time process and decay rate of STM and MTM, we believe this is unlikely. It has been shown that no memory trace is found in *amn* mutant flies 24 h after space training^32^. This could be due to the difference between mutation and conditional knockdown. 2, Our data showed that overexpression of Rac1 damaged the cellular memory trace and forgotten memory. Our findings and other works suggest that learning activates Rac1 to prevent learned information from being further processed. 3, Cdc42 overexpression promotes forgotten memory retrieval. Could this indicate that our protocol retrieved decayed ARM as well? We believe our protocol retrieves mainly ASM but not ARM as follows. A) There was no effect on retrieving forgotten memory when the expression of rad and octβ2R was reduced. B) Rac1 V12 damaged forgotten memory retrieval, suggesting that ASM forgetting is the main form of memory retrieved. C) Cold-shock treatment blocked forgotten memory retrieval and the formation of a cellular memory trace. Then, what causes the enhancement of forgotten memory retrieval in cdc42 V12 flies? We propose two different scenarios. A) Recent studies have demonstrated competition between ARM and LTM^32^. We hypothesize that the establishment and storage of one-cycle aversive conditioning memory, mainly ASM, also competes with ARM. The expression of cdc42 V12 damages ARM maintenance and promotes the memory established in KCαβ. B) Cdc42 also regulates forgotten memory retrievability, for example, preserving the synaptic connectivity between KCαβ and their downstream neurons. It would be of great interest to further dissect the role of cdc42 in regulating the forgetting process. *4*, Dopamine functions in temporally removing memory traces and weakening synaptic connectivity, as discussed earlier.

Our study is reminiscent of memory saving^7,10,78^. However, we think our study is more advanced in many ways. 1) We provided a cellular-based mechanism to explain “memory” decay. We found that the memory trace in MBON-α3 disappeared after one day of one cycle of training but reappeared after weak simulation, suggesting that the memory in those cells disappeared and that those cells could not differentiate between CS+ and CS- stimuli. 2) Upon recalling, the memory trace reappeared in MBON-α3 immediately after stimulation, suggesting that the excitability of cells is changed after training and is beneficial to the next training. 3) We also revealed a negative regulatory mechanism for memory recall. We found that dopaminergic neurons negatively regulate forgotten memory retrieval, suggesting a main inhibitory effect on memory retrieval failure. 4) The so-called “forgotten memory” was found in the brain, in KCαβ.

Currently, the retrievability of active forgetting and the difference between passive and active forgetting in terms of the forgetting process have not been fully experimentally investigated^19^. Our studies compared forgetting behaviors between natural decay (passive forgetting) and interference-based forgetting (active forgetting). We acknowledge that so-called “natural decay” could be a mixture of passive and active forgetting, but the components of active forgetting in natural decay should be less effective than interference-based forgetting. Our data suggested that passive and active forgetting processes could have a similar process but different decay rates. 1, Passive and active forgetting both damage the memory trace in the KCαβ. 2, The forgotten STM could be retrieved in passive and active forgetting, 12 days vs. three days, respectively. 3, It has been suggested that dop1R2 plays an important role in regulating the forgetting process^19,72,79^. Although we do not have direct evidence to show the involvement of dop1R2, our results and previous findings suggest that the retrievability of forgotten memory during passive forgetting is also regulated by the dopamine signal, possibly via dop1R2. 4, The memory trace of both passive and active forgetting could not be observed after the forgotten memory could not be retrieved. Altogether, we hypothesize that passive and active forgetting, dopamine/Rac1 mediated, possess the same forgetting process, and the only difference between these two is the decay rate. Cell activity returning to the base level as an animal loses the stored memory is also observed in complete retrograde amnesia mice^80^.

Compared to studies on the cellular and molecular mechanisms of memory formation, research on forgetting is only in its infancy. By combining behavioral experiments and live-cell imaging to track the formation of the cellular memory trace in fruit flies, our data showed that forgotten memory is preserved and retrievable in most conditions. Our study recharacterized many known “memory proteins” during forgotten memory formation and retrieval. The delicate cellular regulation and complicated molecular gating ensure that memory is retrievable only when the animal needs it. As memory forgetting and caused behavioral damage are observed in many neurological disorders, such as autism and AD^5,81^, our findings further reveal the myth of forgetting and inform future studies on pathological forgetting.

## Methods

### Behavioral analyses

The aversive olfactory paradigm was performed by training approximately 60–100 2-day-old flies in a T-maze as previously described protocols^29^. The two odors used were 3-octanol (OCT) and 4-methylcyclohexanol (MCH). During the training phase, flies received 12 × 1.5 s pulses of 90 V DC electric shock in the presence of the shock-paired odor (CS+) for 60 s, followed by 45 s of fresh air flushing, then exposed them sequentially to the other odor (CS-). All used materials were listed in the Table 1. For testing, flies were transported to a T-maze and given 2 min to choose between the CS+ or CS-. The test was conducted in a temperature-controlled room with 70% relative humidity under dim red light. For each experiment, the one-half performance index (PI) was calculated as the number of flies selecting CS- odor minus the number of flies selecting CS+ odor, divided by the total number of flies. The final PI value is the average score of two complementary experiments with each odor.

For anesthesia-sensitive memory (ASM) and anesthesia-resistant memory (ARM) experiments, flies were transferred into a pre-chilled vial and immersed in ice-cold water for 2 min. The flies were given clod shock 2 h after the conditioning and tested ARM 1 h later at 27 °C.

A retroactive interference paradigm was performed for the interference experiments where flies were conditioned to a novel pair of odors (ethyl acetate and isoamyl acetate) 1.5 h after the initial learning (OCT/MCH). Interference-based forgetting was tested either three days after the initial learning or 8days with OCT and MCH.

### Western blot analysis

Ten heads of flies were homogenized in SDS sample buffer and centrifuged at 16,000 x *g* for 3 min. The supernatant was collected and separated on Tris-tricine gels. Subsequently, the separated proteins were transferred to a nitrocellulose membrane, blocked with 5% nonfat dried milk, and blotted with primary antibodies at four °C overnight. A secondary antibody was applied for 1 h at room temperature, and the signal was visualized with enhanced chemiluminescence.

### Drug feeding

Flies were fed with 35 mM cycloheximide in 5% glucose for blocking protein synthesis 12 h before and 24 h after training. For blocking transcription, flies were fed with actinomycin D 5 µg/ml 12 h before and 24 h after 90 V training, and 24 h before mild retraining in the other group. Actinomycin D was dissolved in DMSO as stock and then diluted to 5 µg/ml in 5% glucose. For labeling newly synthesized peptides, flies were fed with 600 µM puromycin in 5% glucose 12 h before and 24 h after training and then measured levels of new protein synthesis using an anti-puromycin antibody. Control flies were fed with 5% glucose only for the same amount of time.

### Fluorescence microscopy and in vivo calcium imaging

Flies were collected and trained prior to the calcium imaging, following the training paradigm for the behavioral assay mentioned above. We stuck the fly into a trimmed 200 μl tip so that the head of the fly was fixed and exposed for further preparation. Then, the cuticle of the target area was removed using fine tweezers (Dumont#5 500341, World Precision Instruments). Fibers and fat bodies were removed as well for better image acquisition.

Then, the prepared fly was placed in a custom-made chamber on the stage of our upright microscope (BX51WI, Olympus) equipped with a 40x water-immersion objective lens (LUMPlanFLN, Olympus), an excitation illuminator (Hyper S300E, YODN), and a back-illuminated sCMOS camera (pco.edge 4.2bi, PCO). We applied AHL buffer^82^ as the medium between the fly head and water-immersion objective lens. The GCaMP signals were excited by 488 nm light with the intensity adjustment set to 6%. Time-series images were acquired by VisiView® Software (ver. 4.4, VISITRON) at a 5 Hz frame rate.

Air delivery of all the time-series images followed the sequence of 20 s air, 10 s CS+ odor, 30 s air, 10 s CS- odor, and 10 s air, controlled by our custom-made gas control system. The concentration of both odors was the same as those of the behavioral assay. For *UAS-jGCaMP7(f); VT49246-Gal4* flies, we set the bin value to 1 and the exposure time to 19 ms. Whereas the bin value of *G0239-Gal4* > *UAS-jGCaMP7(f)* and *MB630B-Gal4* > *UAS- jGCaMP7(f)* flies were set to 4. And the exposure time of both *G0239-Gal4* > *UAS-jGCaMP7(f)* and *MB630B-Gal4* > *UAS-jGCaMP7(f)* flies was set to 50 ms.

### Calcium imaging data processing and quantification

The processing and quantification of all images were done in Fiji^83^. All collected time-series images went through TrakEM2, the built-in plugin of Fiji, for image registration. Regions of interest were defined manually on all images. The fluorescence of ROIs was divided by the fluorescence of the background of the same size and shape near the ROIs at each time point for normalization. We then calculated ∆F/F_0_, a time-dependent change in relative fluorescence intensity. F_0_ is the average normalized ROI intensity of the frames 5 s prior to odor delivery (t = −5 to 0 s), and ∆F is the subtraction of F_0_ from normalized ROI intensity at each time point. Bar graphs represent the mean of ΔF/F0 during the 10 s odor delivery.

### Statistical analysis

All the raw data were analyzed with GraphPad Prism 6.0 statistical software. Comparison between two groups was evaluated by unpaired *t*-test, and multiple groups were evaluated via one-way analysis of variance (ANOVA). Comparisons between the responses of CS+ and CS- within each group were made by a two-tailed paired *t*-test. Statistical results were presented as means ±S.E.M. The asterisks marked on the bar plots indicate the statistically significant, ^∗^*p* < 0.05; ^∗∗^*p* < 0.01; ^∗∗∗^*p* < 0.001; ^∗∗∗∗^*p* < 0.0001; ns, not significant.

### Reporting summary

Further information on research design is available in the Nature Portfolio Reporting Summary linked to this article.

### Supplementary information

Supplementary Information

Reporting Summary

### Source data

Source Data

## Data Availability

Data are available in the Article, Supplementary Information or Source Data file. Source data are provided with this paper.
