# Supplementary material for: Forgotten memory storage and retrieval in Drosophila
Source: Nat Commun. 2023 Nov 7;14:7153. doi: 10.1038/s41467-023-42753-x (PMC10630420; doi:10.1038/s41467-023-42753-x)
Supplement: Supplementary file 1 — Supplementary Information [file 41467_2023_42753_MOESM1_ESM.pdf]

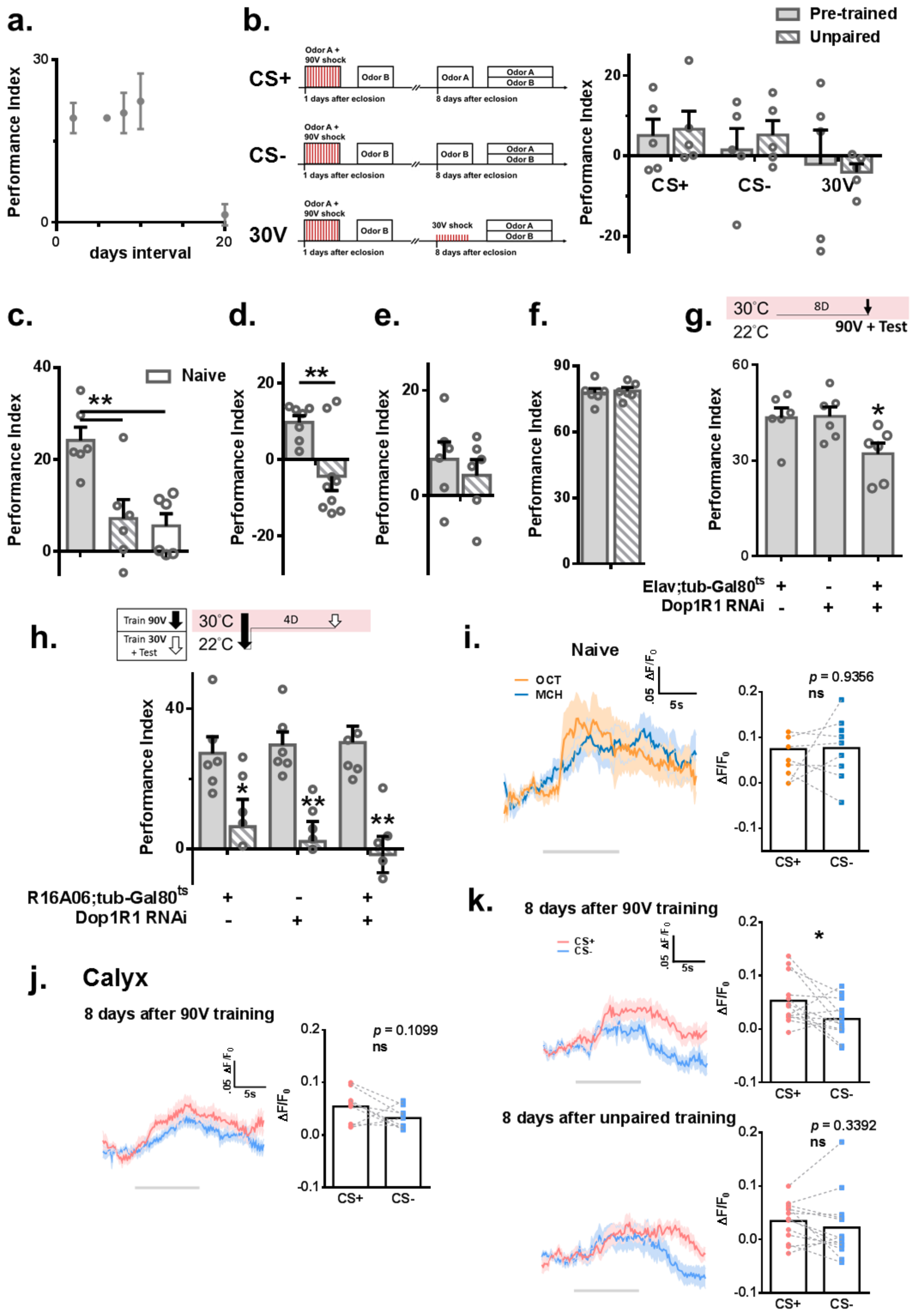

Supplementary Fig. 1. Memory of one cycle aversive conditioning is retrievable.

a) Summary of pre-trained group behavioral performance from figure 1b. **b**) Only CS+ or CS- stimulation or 30V electric shock for 2 minutes would not recall the forgotten memory. N=5 for each group. Statistical comparisons were carried out by

two-tailed paired t-test, p=0.8034, 0.5766 and 0.8211. **c**) Pre-trained flies displayed higher performance than unpaired training flies and naïve flies. N=6 for each group. Statistical comparison was carried out by one-way ANOVA with Dunnett's post-hoc test, p=0.0045 and 0.0022. **d**) The forgotten memory could be established and retrieved in older flies. We 90V trained 8 days after eclosion flies and delivered second 30V mild retraining 5 days later. N=7 and 9. Statistical comparisons were carried out by two-tailed paired t-test, p=0.0075. **e**) The memory performance of pre-trained flies was similar to the unpaired flies, 6 hours after forgotten memory was retrieved. N=6 for both groups. Statistical comparisons were carried out by two-tailed paired t-test, p=0.5011. **f**) There was no difference in 30V electric reactivity between pre-trained and unpaired flies 8 days after. N=6 for both groups. Statistical comparisons were carried out by two-tailed paired t-test, p=0.7340. **g**) Pan-neural expression of Dop1R1 RNAi, drove by elav-gal4, decreased 3 minutes memory performance. N=6 for each group. Statistical comparison was carried out by one-way ANOVA with Dunnett's post-hoc test, p=0.0408. **h**) Knocked down dop1R1 in the γ neurons 4 days before 30V mild retraining would not affect forgotten memory retrieval. N=6 for each group. Statistical comparisons were carried out by two-tailed paired t-test, p=0.0433, 0.0022 and 0.001. **i**) KCαβ of naïve flies responded to OCT and MCH. N=9. Statistical comparisons were carried out by two-tailed paired t-test, p=0.9356. **j**) There was no calcium signal difference between CS+ and CS- in the calyx region after training in VT49246>UAS-GCaMP flies. N=9. Statistical comparisons were carried out by two-tailed paired t-test, p=0.1099. **k**) Increased calcium signal from GCaMP7f was observed in the pre-trained group with MCH as CS+ and OCT as CS-. N=13 for both groups. Statistical comparisons were carried out by two-tailed paired t-test, p=0.0390 and 0.3392.*p<0.05. **p<0.01. ***p<0.001. In all figures, each value represents mean ± SEM.

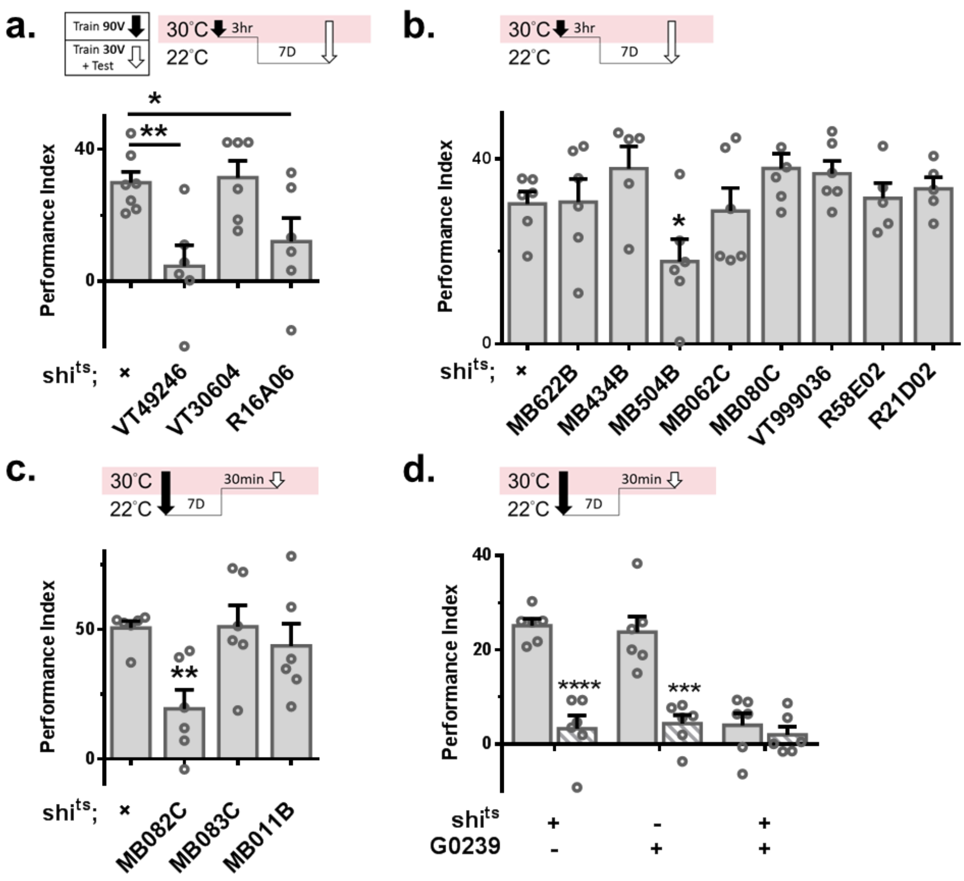

Supplementary Fig. 2. Neural circuit involved in forming and retrieval forgotten memory.

a) Output activity inhibition in γ neurons, αβ neurons during 90V training and 3 hours after 90V training affected forgotten memory formation. N=7, 6, 6, 6. Statistical comparison was carried out by two-tailed unpaired t-test, p=0.0036, 0.0362. **b**) Output activity inhibition in PPL1 neurons (MB504B) during 90V training and 3 hours after 90V training affected forgotten memory formation. N= 6, 6, 5, 6, 6, 6, 6, 5, 5. Statistical comparison was carried out by two-tailed unpaired t-test, p=0.0468. **c** and **d**) Output activity inhibition in MBON-α3 (MB082C and G0239) during 30V mild retraining affected forgotten memory retrieval. N=6 for each group. Statistical comparison was carried out by two-tailed unpaired t-test. For figure c, p=0.0026. For figure d, p<0.0001, p=0.0004, 0.5188. *p<0.05. **p<0.01. ***p<0.001. ****p<0.0001.

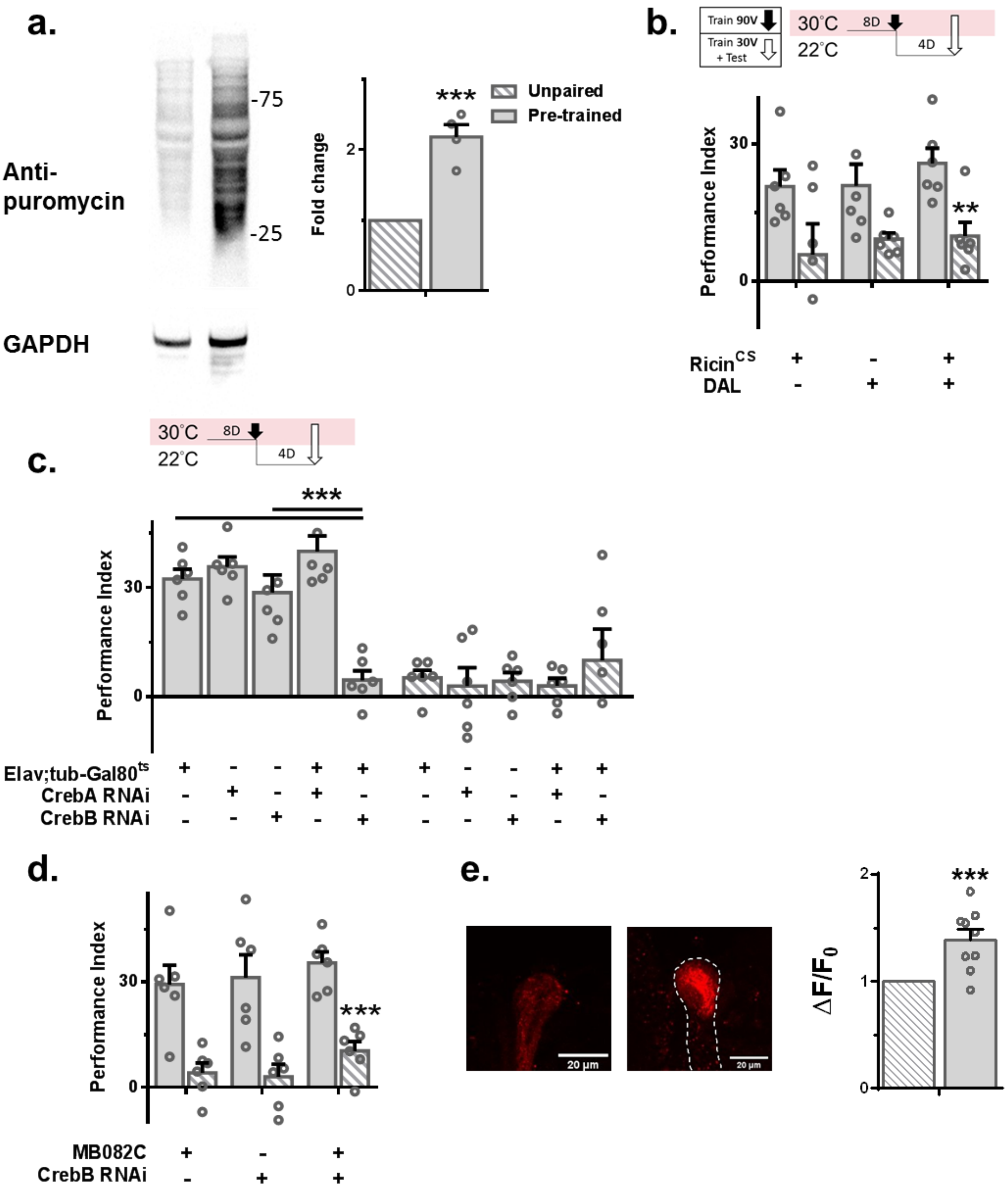

a.
Anti-puromycin
GAPDH
-75
-25
Fold change
***
Unpaired
Pre-trained
b.
Train 90V
Train 30V + Test
30°C
22°C
8D
4D
Performance Index
**
Ricin^{CS}
DAL
c.
Elav;tub-Gal80^{ts}
CrebA RNAi
CrebB RNAi
d.
MB082C
CrebB RNAi
e.
20 µm
$\Delta F/F_0$

Supplementary Fig.3. One cycle aversive conditioning triggers protein synthesis that is for forgotten memory formation.

a) Flies were fed with puromycin 12 hours before and 24 hours after 90V training. 24 hours after training flies head was collected for western blot analysis. Left, reprehensive western result. Right, statistic results. N=4 for each group. Statistical comparison was carried out by two-tailed unpaired t-test, p=0.0005. **b**) Inhibited protein synthesis activity in *DAL-Gal4>ricin*^{cs} flies would not affect forgotten memory formation. N=6 for each group. Statistical comparison was carried out by two-tailed unpaired t-test, p=0.0053. **c**) Knocked down CrebB but CrebA in *Elav-Gal4>ricin*^{cs} flies under Gal80^{ts} regulation abolished the forgotten memory. Adult transgenic flies were transferred to the 30°C environment for 8 days before 90V training. 30V mild retraining was delivered 4 days later. N=6 for each group. Statistical comparison was carried out by one-way ANOVA with Dunnett's post-hoc test, p=0.0001, 0.0005. **d**) Knocked down CrebB in MBON-α3 neurons would not affect forgotten memory. N=6 for each group. Statistical comparison was carried out by two-tailed unpaired t-test, p=0.0001. **e**) More RFP signals were found in α3 compartment 24 hours after 90V training. Left, representatives imagine. Right, statistic results. N=9 for each group. Statistical comparison was carried out by two-tailed unpaired t-test, p=0.0008. **p<0.01. ***p<0.001. In all figures, each value represents mean ± SEM.

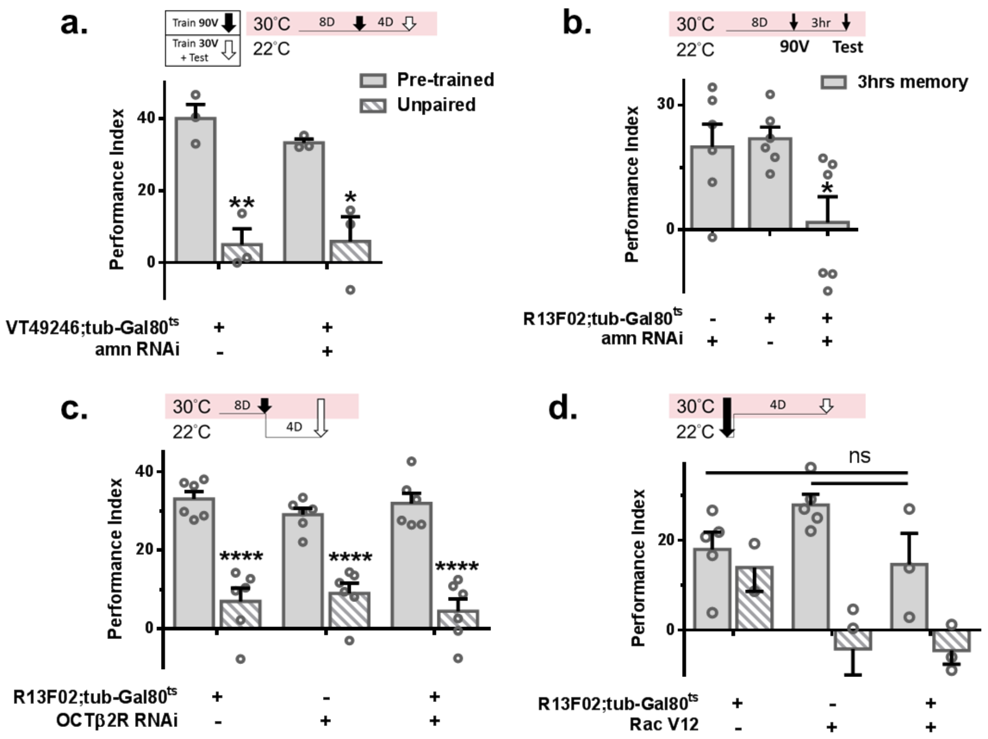

Supplementary Fig.4. Unconsolidated memory is the main component to form forgotten memory.

a) Knocked down amn in adult αβ neurons did not affect forgotten memory formation. The mild retraining was given 4 days after 90V training. N=3 for each group. Statistical comparison was carried out by two-tailed unpaired t-test, p=0.0039, 0.0164. **b**) Knocked down amn in adult MBNs for 8 days reduced 3-h memory expression. N=6 for each group. Statistical comparison was carried out by one-way ANOVA with Dunnett's post-hoc test, p=0.0231. **c**) Knocked down octβ2R in adult MBNs did not affect forgotten memory formation. The mild retraining was given 4 days after 90V training. N=6 for each group. Statistical comparison was carried out by two-tailed unpaired t-test, p<0.0001, p<0.0001, p<0.0001. **d**) Overexpression Rac1 V12 in adult MBNs 4 days before mild retraining did not affect forgotten memory retrieval. N=5, 2, 5, 3, 3, 3. Statistical comparison was carried out by one-way ANOVA with Dunnett's post-hoc test, p=0.7915, 0.0918. *p<0.05. **p<0.01. ****p<0.0001. In all figures, each value represents mean ± SEM.

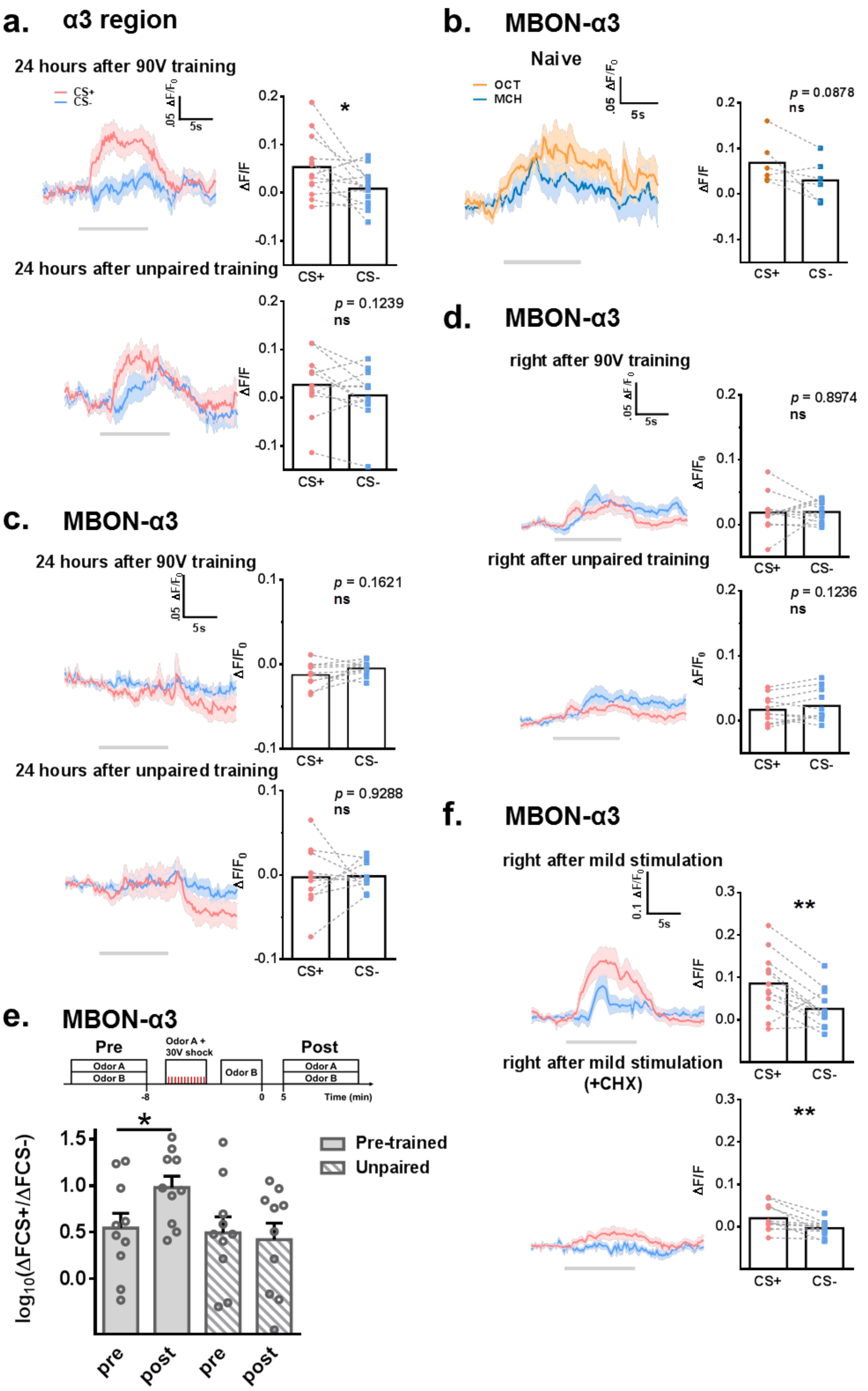

a.
α3 region
24 hours after 90V training
CS+
CS-
.05 ΔF/F0
5s
ΔF/F
*
CS+
CS-
24 hours after unpaired training
p = 0.1239
ns
b.
MBON-α3
Naive
OCT
MCH
p = 0.0878
ns
c.
MBON-α3
24 hours after 90V training
p = 0.1621
ns
ΔF/F0
24 hours after unpaired training
p = 0.9288
ns
d.
MBON-α3
right after 90V training
p = 0.8974
ns
right after unpaired training
p = 0.1236
ns
e.
MBON-α3
Pre
Odor A + 30V shock
Post
Odor A
Odor B
Time (min)
log10(ΔFCS+/ΔFCS-)
Pre-trained
Unpaired
pre
post
f.
MBON-α3
right after mild stimulation
0.1 ΔF/F0
**
right after mild stimulation (+CHX)

Supplementary Fig.5. Cellular memory trace formed in the α3 region of αβ neurons and MBON-α3 after training.

a) Increased calcium signal after CS+ odor exposure in the α3 region of αβ neurons 24 hours after 90V training. N=13 and 12(top to bottom). Statistical comparisons were carried out by two-tailed paired t-test, $p=0.0292$ and 0.1239. **b**) MBON-α3 of naïve flies responded to OCT and MCH. N=6. Statistical comparisons were carried out by two-tailed paired t-test, $p=0.0878$. **c**) There was no observed increased calcium signal after CS+ odor exposure in MBON-α3 24 hours after 90V training. N=10 for both groups. Statistical comparisons were carried out by two-tailed paired t-test, $p=0.1621$ and 0.9288. **d**) There was no observed increased calcium signal after CS+ odor exposure in MBON-α3 right after 90V training. N=12 and 11(top to bottom). Statistical comparisons were carried out by two-tailed paired t-test, $p=0.8974$ and 0.1236. **e**) CS+/CS- calcium response ratio was higher after mild retraining in MBON-α3 in pre-trained flies. N=10 for both groups. Statistical comparisons were carried out by two-tailed paired t-test, $p=0.0448$ and 0.7771. **f**) CHX treatment 24 hours before mild retraining decreases the formation of cellular memory trace in MBON-α3 right after mild retraining. N=12 for both groups. Statistical comparisons were carried out by two-tailed paired t-test, $p=0.0028$ and 0.0064. $^{*}p<0.05$. $^{**}p<0.01$. Calcium imaging data ($\Delta F/F0$) was evaluated by two-tailed paired t-test. In all figures, each value represents mean ± SEM. The N values in **a**, **b**, **c**, **d**, and **f**) represent the number of flies recorded in each experiment, whereas the N value in **e**) represents the batches of flies trained and tested in the behavioral assay.

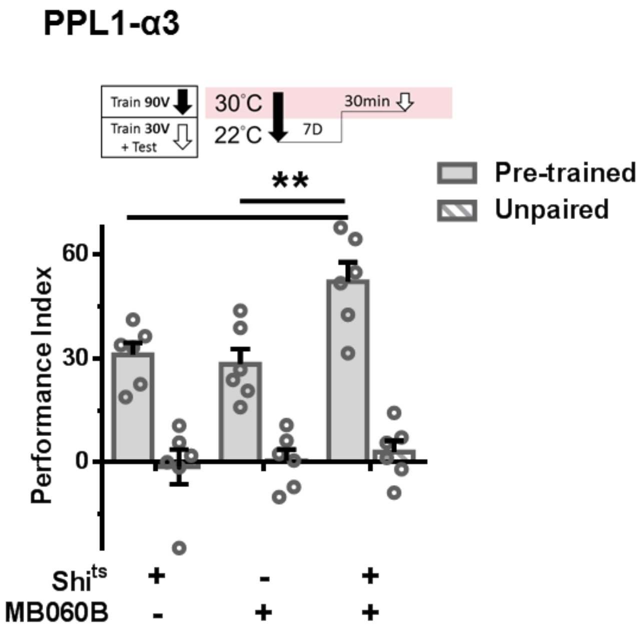

Supplementary Fig. 6. Reduced PPL1-α3 activity promotes forgotten memory retrieval.

Output activity inhibition of PPL1-α3 (MB060B) during mild retraining promoted forgotten memory retrieval. N=6 for each group. Statistical comparison was carried out by one-way ANOVA with Dunnett's post-hoc test, $p=0.0095$, 0.0040. **$p<0.01$. In all figures, each value represents mean ± SEM.

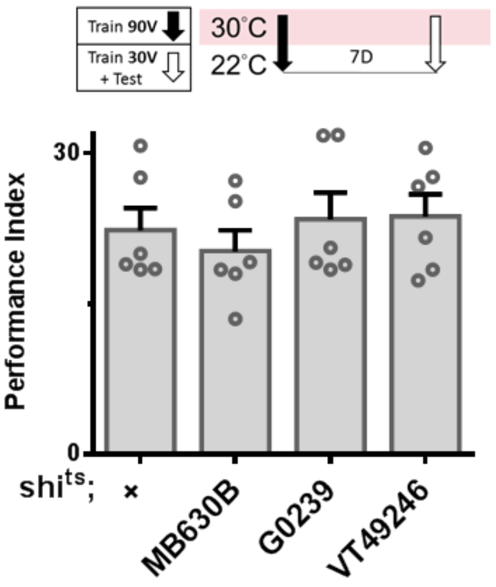

Supplementary Fig. 7. No behavioral deficit found in the permissive temperature

There was no observed retrieved forgotten memory defect in *MB630B-Gal4>UAS-shi^{ts}*, *G0239-Gal4>UAS-shi^{ts}*, *VT49246-Gal4>UAS-shi^{ts}* flies cultured in permissive temperature. N=6 for each group. Statistical comparison was carried out by two-tailed unpaired t-test. p=0.5026, 0.7624, 0.6747. In all figures, each value represents mean ± SEM.
