# Supplementary material for: Forgotten memory storage and retrieval in Drosophila
Source: Nat Commun. 2023 Nov 7;14:7153. doi: 10.1038/s41467-023-42753-x (PMC10630420; doi:10.1038/s41467-023-42753-x)
Supplement: Supplementary file 2 — Reporting Summary [file 41467_2023_42753_MOESM2_ESM.pdf]

nature portfolio

Corresponding author(s): HsuehCheng Chiang

Last updated by author(s): Sep 27, 2023

Reporting Summary

Nature Portfolio wishes to improve the reproducibility of the work that we publish. This form provides structure for consistency and transparency in reporting. For further information on Nature Portfolio policies, see our Editorial Policies and the Editorial Policy Checklist.

Statistics

For all statistical analyses, confirm that the following items are present in the figure legend, table legend, main text, or Methods section.

n/a	Confirmed	
☐	☒	The exact sample size (*n*) for each experimental group/condition, given as a discrete number and unit of measurement
☐	☒	A statement on whether measurements were taken from distinct samples or whether the same sample was measured repeatedly
☐	☒	The statistical test(s) used AND whether they are one- or two-sided<br>*Only common tests should be described solely by name; describe more complex techniques in the Methods section.*
☒	☐	A description of all covariates tested
☐	☒	A description of any assumptions or corrections, such as tests of normality and adjustment for multiple comparisons
☐	☒	A full description of the statistical parameters including central tendency (e.g. means) or other basic estimates (e.g. regression coefficient) AND variation (e.g. standard deviation) or associated estimates of uncertainty (e.g. confidence intervals)
☐	☒	For null hypothesis testing, the test statistic (e.g. *F*, *t*, *r*) with confidence intervals, effect sizes, degrees of freedom and *P* value noted<br>*Give P values as exact values whenever suitable.*
☒	☐	For Bayesian analysis, information on the choice of priors and Markov chain Monte Carlo settings
☒	☐	For hierarchical and complex designs, identification of the appropriate level for tests and full reporting of outcomes
☒	☐	Estimates of effect sizes (e.g. Cohen's *d*, Pearson's *r*), indicating how they were calculated

Our web collection on statistics for biologists contains articles on many of the points above.

Software and code

Policy information about availability of computer code

Data collection	Flies were imaged with an upright microscope (BX511WI, Olympus) equipped with 40x water-immersion objective (LUMPlanFLN, Olympus) using 488nm excitation laser. The GFP signals were receive by back-illuminated sCMOS camera (pco.edge 4.2 bi, PCO) attached to our microscope. And the image was acquired by VisiView® Software (ver. 4.4, VISITRON).
Data analysis	Statistical analyses were performed in GraphPad Prism6.01 software. For the imaging data, two-tailed unpaired t-test was used to compare the responses to teacher’s odor between groups. Comparisons between the responses of CS+ and CS- within each group were done by two-tailed

For manuscripts utilizing custom algorithms or software that are central to the research but not yet described in published literature, software must be made available to editors and reviewers. We strongly encourage code deposition in a community repository (e.g. GitHub). See the Nature Portfolio guidelines for submitting code & software for further information.

Data

Policy information about availability of data

All manuscripts must include a data availability statement. This statement should provide the following information, where applicable:

- Accession codes, unique identifiers, or web links for publicly available datasets
- A description of any restrictions on data availability
- For clinical datasets or third party data, please ensure that the statement adheres to our policy

Data are available in the Article, Supplementary Information or Source Data file. Source data are provided with this paper.

Human research participants

Policy information about studies involving human research participants and Sex and Gender in Research.

Reporting on sex and gender	N/A
Population characteristics	N/A
Recruitment	N/A
Ethics oversight	N/A

Note that full information on the approval of the study protocol must also be provided in the manuscript.

Field-specific reporting

Please select the one below that is the best fit for your research. If you are not sure, read the appropriate sections before making your selection.

☒ Life sciences ☐ Behavioural & social sciences ☐ Ecological, evolutionary & environmental sciences

For a reference copy of the document with all sections, see nature.com/documents/nr-reporting-summary-flat.pdf

Life sciences study design

All studies must disclose on these points even when the disclosure is negative.

Sample size	Sample sizes were not predetermined and no statical method was used to determine the sample size. Exact sample numbers were described in each figures for each genotype. For behavioral experiences, most sample size were around or more than 6.<br>For image quantitative experiments, figure 1e, 2c, 4b, 5, 6d, 6e, 6f has sample size equal to or more than 10.<br>Sample size based on our previous and other experiments over the past years. (Ge et al., 2004 PNAS, Shuai et al., 2010 Cell, Chen et al, 2019 Aging Cell, Zhao et al., 2021 Elife)
Data exclusions	No data was excluded
Replication	All replication were repeated on different days with different batch of flies.
Randomization	Same genotypes were randomly allocated to experimental groups.
Blinding	Not blind to experimenters. However, different behavioral experiments were performed by different experimenters and the conclusion of results are supporting each other. Instead, we relied upon independent experimenter analysis and similar experiment to confirm the results.

Reporting for specific materials, systems and methods

We require information from authors about some types of materials, experimental systems and methods used in many studies. Here, indicate whether each material, system or method listed is relevant to your study. If you are not sure if a list item applies to your research, read the appropriate section before selecting a response.

Materials & experimental systems

n/a	Involved in the study
☐	☒ Antibodies
☒	☐ Eukaryotic cell lines
☒	☐ Palaeontology and archaeology
☐	☒ Animals and other organisms
☒	☐ Clinical data
☒	☐ Dual use research of concern

Methods

n/a	Involved in the study
☒	☐ ChIP-seq
☒	☐ Flow cytometry
☒	☐ MRI-based neuroimaging

Antibodies

Antibodies used	Mouse monoclonal anti-puromycin, 1:200, PMY-2A4<br>Peroxidase AffiniPure Goat Anti-Mouse IgG (H+L), 1:2000, 115-035-003<br>Rabbit anti-GAPDH, 1:50000, GTX100118
Validation	DSHB (AB_2619605), https://dshb.biology.uiowa.edu/PMY-2A4<br>Jackson ImmunoResearch (115-035-003), https://www.jacksonimmuno.com/catalog/products/115-035-003<br>GeneTex (GTX100118), https://www.genetex.com/Product/Detail/GAPDH-antibody/GTX100118

Animals and other research organisms

Policy information about studies involving animals; ARRIVE guidelines recommended for reporting animal research, and Sex and Gender in Research

Laboratory animals	Drosophila melanogaster wild type and all transgenic strains used is provided in methods.
Wild animals	We did not use wild animals
Reporting on sex	All male and female flies were mixed for experiment. There were no pre-selection.
Field-collected samples	Did not involve collection in the field
Ethics oversight	Was no required for experiments on invertebrates.

Note that full information on the approval of the study protocol must also be provided in the manuscript.
